# Syndrome differentiation and treatment of pediatric central precocious puberty: role of the spleen-kidney axis

**DOI:** 10.3389/fimmu.2026.1900121

**Published:** 2026-08-25

**Authors:** Yujun Luo, Chaojie Huang, Yanting Lu, Jieying He, Hailing Chen, Jinghua Yang

**Affiliations:** 1 Pediatrics Department, Guangdong Provincial Hospital of Chinese Medicine, Guangzhou, Guangdong, China; 2 The Second Clinical College of Guangzhou University of Chinese Medicine, Guangzhou, Guangdong, China

**Keywords:** central precocious puberty, dual regulation of the kidney and spleen, hypothalamic-pituitary-gonadal axis, immune-inflammatory regulation, TCM syndrome differentiation

## Abstract

Pediatric central precocious puberty (CPP) is driven by premature activation of the hypothalamic-pituitary-gonadal (HPG) axis, along with immune-inflammatory imbalance, metabolic dysfunction, obesity, and gut microbiota-derived signals. According to traditional Chinese medicine (TCM) principles, kidney yin deficiency with hyperactive ministerial fire and spleen deficiency with phlegm-dampness form the basis for central pubertal activation and peripheral metabolic-inflammatory burden, thus supporting a combined approach for obesity-related or mixed-pattern CPP. Representative prescriptions include modified Zhibai Dihuang pills with Liujunzi decoction and modified Danzhi Xiaoyao powder with Erchen decoction, which integrate kidney yin-nourishment and ministerial fire-clearance with spleen qi-strengthening and phlegm resolution. Available clinical evidence suggests potential benefits in controlling secondary sexual characteristics, reducing gonadotropin and sex-steroid levels, and slowing bone-age advancement. Mechanistic studies indicate possible regulation of kisspeptin/GPR54/GnRH and LH/FSH-sex-steroid signaling, Th1/Th2/Treg homeostasis, NF-κB/NLRP3 pathways, adipokine imbalance, insulin resistance, hypothalamic inflammation, and gut microbiota-associated metabolism. However, most mechanistic links remain indirect, comparative trials are scarce, and direct correction of pathogenic MKRN3 or DLK1 variants has not been demonstrated. In this review, we have discussed the role of kidney and spleen dysfunction in CPP and explored the underlying mechanisms – particularly in the context of the neuro-endocrine-immune network that links central neuroendocrine activation with peripheral immune-metabolic signals. In addition, the application of TCM in the management of CPP, and the translational challenges of applying TCM as an adjunct to established CPP management have also been explored. Future research should prioritize CPP-specific pathway validation, identification of syndrome biomarkers, multi-omics-based stratification, multicenter trials, and long-term pediatric safety monitoring.

## Graphical Abstract

**Figure:**
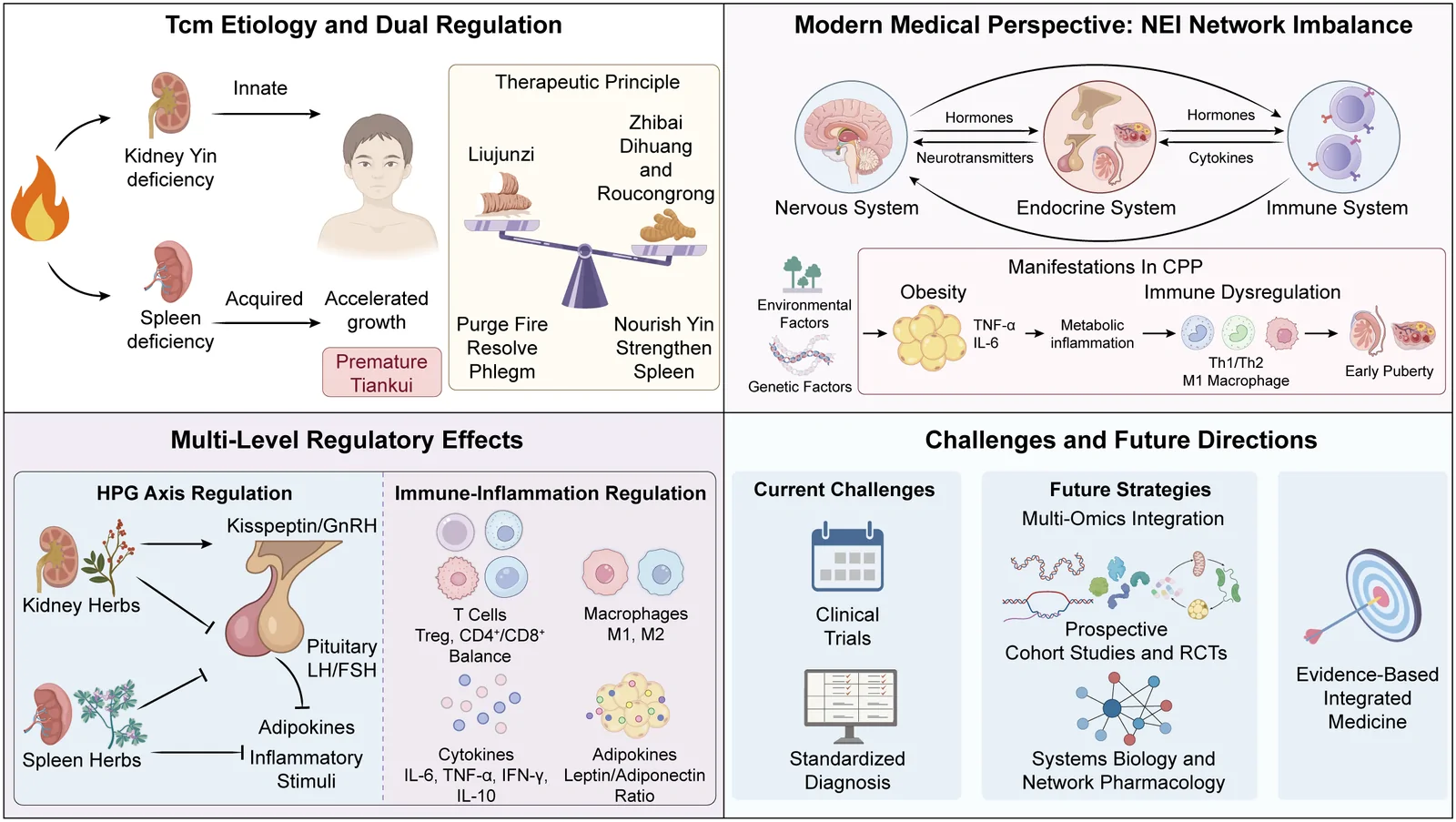
Integrated traditional Chinese medicine (TCM) and biomedical framework for kidney–spleen dual regulation in pediatric central precocious puberty (CPP). The TCM framework of kidney yin deficiency and spleen deficiency with phlegm-dampness is conceptually linked to neuro-endocrine-immune (NEI) network imbalance, premature hypothalamic–pituitary–gonadal (HPG) axis activation, and immune-metabolic dysfunction. Kidney–spleen dual regulation may modulate these processes through multilevel neuroendocrine and immune-inflammatory mechanisms. Standardized diagnosis, multi-omics integration, clinical trials, and long-term safety evaluation remain key priorities.

## 1 Introduction

Central precocious puberty (CPP) is a pediatric endocrine disorder characterized by the premature appearance of secondary sexual characteristics due to early activation of the hypothalamic-pituitary-gonadal (HPG) axis. Affected children may experience rapid linear growth, accelerated skeletal maturation, compromised adult height, and potential psychosocial and longer-term health consequences (1). Pubertal initiation is governed by coordinated excitatory and inhibitory neuroendocrine signals rather than a single endocrine event. In particular, the kisspeptin-GPR54 system stimulates pulsatile gonadotropin-releasing hormone (GnRH) secretion and activates the HPG axis (2, 3). While this endocrine switch may serve as the key driver of CPP, it does not fully explain the marked variation in age at onset, progression, or metabolic phenotypes among the affected children.

CPP shows considerable etiological heterogeneity. Loss-of-function variants in MKRN3 are a well-established monogenic cause; since MKRN3 is maternally imprinted and paternally expressed, pathogenic variants that cause CPP are transmitted paternally (4, 5). Hypothalamic MKRN3 expression is high before puberty and decreases as puberty approaches, which is consistent with reduced GnRH secretion (5, 6). In addition, abnormalities in the DLK1 gene have been linked to endocrine disruption, excess adiposity, metabolic dysfunction, and gut dysbiosis, which may influence pubertal timing through neuroendocrine, metabolic, and immune-inflammatory pathways (1, 7–9). Furthermore, increased hypothalamic expression of the N6-methyladenosine (m6A) RNA demethylase FTO reduces m6A modification of Bdnf mRNA and enhances BDNF/PI3K/Akt/GnRH signaling in an experimental model, although no causal FTO variant has been established as a monogenic cause of human CPP (10).

Recent epidemiological evidence underscores the clinical and public health relevance of CPP. A global systematic review and meta-analysis reported survey-based pooled prevalence estimates of 7.87% in girls and 3.98% in boys. Registry-based estimates of CPP vary widely, with prevalence rates ranging from 37 to 935.1 per 100,000 girls and from 0.46 to 37.4 per 100,000 boys, and incidence rates ranging from 1.123 to 489.3 per 100,000 girls and from 0.096 to 22.4 per 100,000 boys (11). In China, a school-based study reported an adjusted prevalence of 6.19%, with a higher prevalence in girls than in boys (11.47% vs. 3.26%) (12). A subsequent multicenter prospective cohort of 8025 children reported an adjusted incidence of 1.7%, with 2.9% among girls and 1.1% among boys (13). Although estimates vary by population and study design, these data demonstrate a substantial and heterogeneous disease burden. Temporal trends and the increasing burden of childhood obesity further confound the epidemiological patterns of CPP. Most studies included in a recent scoping review reported an increase in precocious puberty during the COVID-19 pandemic, particularly among girls; reduced physical activity, increased screen exposure, altered diet, sleep disruption, and weight gain were considered potential contributors (14). Chinese cohort data likewise show a greater frequency of early or precocious puberty among girls who are overweight or obese (12, 13). In addition, a hospital-based case-control study reported a close association between prolonged state of obesity in early childhood with increased odds of CPP, especially in girls (15). Furthermore, the prevalence of obesity among Chinese school-age children increased from 15.5% in 2010 to 24.2% in 2019, and 29.4% in 2022 (16). Therefore, CPP should be considered not only an endocrine condition but also a developmental disorder affected by metabolic and environmental factors.

According to traditional Chinese medicine (TCM), CPP mainly corresponds to the categories of “Ruli” (premature thelarche) and “Yuejing Zaochao” (premature menarche). Its pathogenesis is commonly interpreted through the principles that the kidney governs growth and reproduction and that the spleen is the acquired foundation of the body (17, 18). Kidney yin deficiency may permit hyperactivity of ministerial fire and the premature arrival of Tiankui, whereas impaired spleen transportation and transformation may generate phlegm-dampness and disturb the Chong and Ren meridians. These processes are frequently intertwined rather than mutually exclusive. Accordingly, simultaneous nourishment of kidney yin and clearance of ministerial fire with strengthening of spleen qi and resolution of phlegm-dampness provides a syndrome-based rationale for managing premature neuroendocrine activation and metabolic or constitutional abnormalities.

From a contemporary biomedical perspective, the rationale for kidney-spleen dual regulation can be explored through the neuro-endocrine-immune (NEI) network, wherein neural, endocrine, immune, and metabolic signals interact bidirectionally. Obesity-related CPP may involve adipokine imbalance, hypothalamic inflammation, and altered neuroendocrine signaling (7, 8, 19), whereas the gut microbiota may connect environmental exposure and nutrient metabolism with immune activity and reproductive endocrine regulation (7). Therefore, the kidney-tonifying and spleen-invigorating prescriptions may influence HPG-axis activity, inflammatory cytokine release, immune-cell balance, insulin sensitivity, microbial metabolism, and neuroimmune communication (20, 21). Nevertheless, the “organs” in TCM are not direct anatomical equivalents but rather interpretive models whose proposed biological correlates require pathway-specific validation. Furthermore, in the context of CPP, there is no evidence that TCM prescriptions for regulating kidney-spleen functions influence MKRN3 or DLK1 variants or imprinting defects or directly normalize FTO-dependent m6A demethylation. Therefore, the effects of these prescriptions mainly target downstream phenotypes and pathway networks rather than the genetic or epigenetic drivers. The current literature largely emphasizes conventional CPP pathogenesis or the pooled clinical effects of TCM, with limited differentiation among kidney-centered, spleen-centered, and dual-regulation strategies. In addition, the efficacy of the formulations is mainly evaluated based on secondary sexual characteristics, hormone levels, or bone-age indices, whereas the associations among treatment response, TCM syndrome patterns, obesity status, and immune-metabolic phenotypes are seldom examined. Likewise, mechanistic studies often discuss the HPG axis, cytokines, adipokines, gut microbiota, and hypothalamic inflammation as parallel topics rather than as interacting components of an NEI network. Moreover, conclusions are frequently extrapolated from network pharmacology, non-CPP disease models, or short-term studies, leaving causal validation, comparative effectiveness, objective syndrome biomarkers, and long-term pediatric safety insufficiently resolved.

To address these gaps, this review traces the transition of CPP pathogenesis from a predominantly kidney-centered model to phenotype-oriented kidney-spleen dual regulation, constructs an NEI framework linking kisspeptin/GPR54/GnRH and pituitary-gonadal signaling with immune-inflammatory pathways, adipokine imbalance, metabolic stress, and microbiota-derived signals, distinguishes CPP-specific evidence from network-level hypotheses and identifies potential mechanisms, and integrates syndrome standardization, biomarker development, multi-omics research, clinical evaluation, and pediatric safety into a unified translational agenda. The objective of this review is to define verifiable mechanisms, identify clinically relevant research priorities, and support evidence-based integration of TCM as an adjunct to established CPP management (Graphical Abstract).

## 2 TCM theory of the etiology and pathogenesis of CPP

### 2.1 Kidney and spleen dysfunction in the onset of CPP

**Figure 1:**
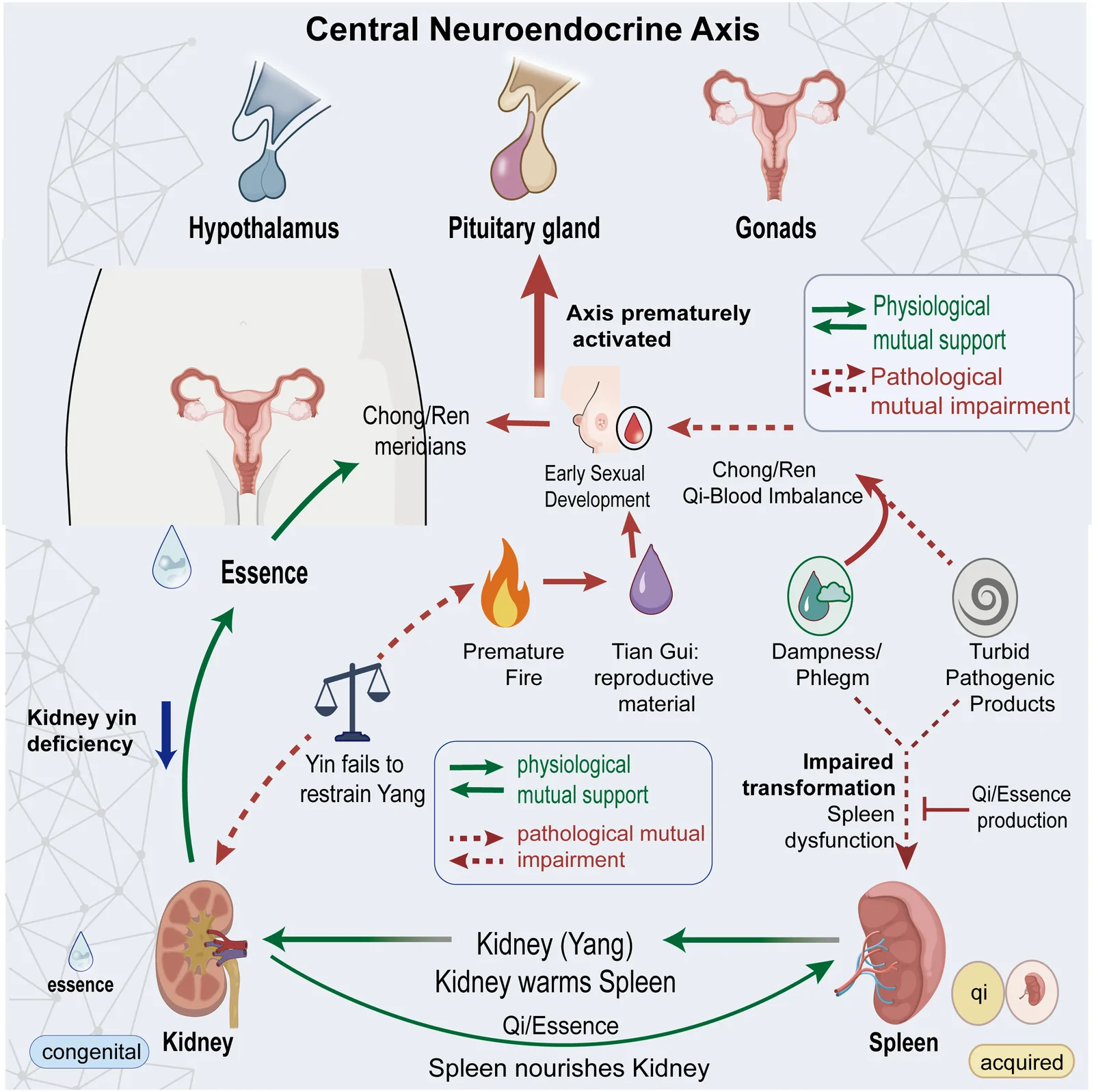
Kidney-spleen dysfunction and premature activation of the central neuroendocrine axis in pediatric CPP. Under physiological conditions, spleen-derived qi and essence replenish kidney essence, while kidney yang supports splenic transportation and transformation; kidney essence, in turn, sustains the Chong and Ren meridians. Kidney-yin deficiency may fail to restrain yang, generating deficient fire, premature emergence of Tiankui, and early sexual development. Concurrent splenic dysfunction promotes phlegm-dampness and turbid products, disrupts Chong/Ren qi-blood, and aggravates kidney-spleen disequilibrium. These convergent processes are proposed to facilitate premature activation of the HPG-axis. Solid green arrows denote physiological mutual support, solid red arrows denote downstream pathological activation, dashed red arrows denote pathological propagation or reciprocal impairment, and blunt-ended lines indicate inhibition. CPP, central precocious puberty; HPG, hypothalamic-pituitary-gonadal.

Kidney and spleen dysfunction are the pathological hallmarks in the development of CPP and may contribute to the premature activation of the central neuroendocrine axis (**Figure 1**). In TCM theory, the kidney is regarded as the congenital foundation of the body as it stores essence and governs growth, reproduction, and development. Given the natural kidney insufficiency in children, congenital weakness or improper postnatal care may lead to kidney yin deficiency. When yin fails to control yang, ministerial fire may become hyperactive. This deficient internal fire can promote the premature arrival of Tiankui, which is closely related to reproductive development according to TCM. As a result, children may show early sexual signs such as breast development and premature menstruation. This explanation is consistent with the modern medical view that CPP is mainly caused by premature activation of the HPG axis and increased pulsatile secretion of GnRH, which also underscores the important role of the kidney in controlling growth and development (22). The spleen is also not fully developed in children, and its functions are relatively weak. Improper diet, especially excessive intake of greasy, sweet, rich, or nourishing foods, may cause damage to the spleen and stomach. The inability of the spleen to effectively transport and transform nutrients can result in the accumulation of water-dampness, eventually leading to phlegm-dampness. At the same time, abnormal nutrient transformation may produce turbid pathological substances. The combination of phlegm-dampness and turbid factors may disturb the Chong and Ren meridians, thereby promoting premature sexual development. Therefore, the strength or weakness of spleen function may influence CPP by affecting nutrient metabolism and formation of pathological substances (22).

The functions of the kidneys and spleen are closely connected in both physiological and pathological settings. Under normal conditions, the spleen transforms food into nutrients and continuously supports kidney essence, which is described as “the acquired foundation nourishing the congenital foundation” in TCM. Likewise, kidney yang warms the spleen and aids in its normal functioning, a phenomenon known as “the congenital foundation supporting the acquired foundation”. Therefore, the kidneys and spleen work synergistically to maintain normal growth and development in children. When this balance is disturbed, kidney deficiency may combine with spleen weakness, or long-term spleen deficiency may further affect the kidney. For example, prolonged kidney yin deficiency may fail to nourish spleen yin, causing poor nutrient transportation and transformation. Similarly, long-term spleen deficiency may reduce the production of qi and blood, which then fails to replenish kidney essence. Therefore, CPP often presents as a mixed condition involving both root deficiency and superficial excess. Clinically, children may show abnormal growth and development, weak constitution, poor appetite, phlegm-dampness retention, and other related symptoms. Studies have suggested that many chronic and complex diseases involve several zang fu organs and body systems, and TCM organ dysfunction may partly correspond to imbalances in neural, endocrine, and immune networks (22). This indicates that simultaneous regulation of the kidney and spleen in children with CPP can restore both physiological balance and NEI network homeostasis, thereby achieving a more comprehensive therapeutic effect.

### 2.2 Dual regulation of the kidney and spleen in CPP

As discussed above, the development of CPP is mainly associated with kidney yin deficiency and hyperactivity of ministerial fire, and formation of phlegm-dampness in the weak spleen (23). Therefore, TCM-based treatment of CPP combines nourishing kidney yin, clearing ministerial fire, strengthening spleen transportation, and removing phlegm-dampness. This holistic approach helps regulate both the congenital and acquired foundations and supports the recovery of yin yang balance, qi-blood harmony, and overall body coordination. Modern medical studies also support the biological importance of kidney-spleen regulation. For example, osteoporosis patients with spleen-kidney yang deficiency exhibit unique intestinal microbiota and metabolic features (24). These findings provide a scientific basis for using dual kidney-spleen regulation in CPP, especially through regulation of the neuro-endocrine-immune network.

Earlier TCM treatment for pediatric precocious puberty was kidney-oriented, since premature sexual development was mainly attributed to kidney yin deficiency, internal deficiency fire, and the premature arrival of Tiankui. Therefore, formulas derived from Liuwei Dihuang pills, Zhibai Dihuang pills, Dabuyin pills, and other yin-nourishing and fire-purging prescriptions were widely used to restrain excessive HPG axis activation. Recently, a meta-analysis and network pharmacology study of nourishing-yin and purging-fire therapy for CPP showed that these treatment approaches can improve clinical efficacy, reduce secondary sexual indicators, decrease serum FSH, LH, and E2 levels, and slow bone-age maturation. The Zhimu-Huangbai herb pair was identified as a core combination in this treatment strategy (25). However, this single therapeutic direction is most suitable for children whose dominant clinical pattern is yin deficiency with fire hyperactivity, such as tidal fever, night sweating, irritability, red tongue, and rapid progression of secondary sexual characteristics. With changes in diet, lifestyle, and the increasing burden of childhood obesity, the role of spleen dysfunction in CPP has gradually received more attention. Spleen-strengthening and phlegm-resolving therapy is prescribed for children with phlegm-dampness accumulation, tendency for being overweight or obese, abdominal fullness, poor appetite, greasy tongue coating, insulin resistance, adipokine imbalance, or metabolic inflammation. A spleen-centered strategy may be particularly relevant to obesity-related CPP because adipose-tissue dysfunction, leptin-adiponectin imbalance, insulin resistance, and chronic low-grade inflammation are known to stimulate the hypothalamic reproductive center (26–31). Nevertheless, when central HPG axis activation, accelerated bone-age, elevated gonadotropins, and typical yin-deficiency fire signs are already prominent, spleen-strengthening and phlegm-resolving therapy alone may not be sufficient to manage the neuroendocrine imbalance associated with CPP. Dual regulation of the kidney and spleen combines the endocrinal advantages of nourishing yin and purging fire with the metabolic-immune advantages of spleen strengthening and phlegm resolution. This approach may be more suitable for children with mixed patterns, such as kidney yin deficiency complicated by spleen deficiency and phlegm-dampness, or obesity-related CPP accompanied by internal heat and early HPG axis activation. Cross-comparative evidence remains limited because few clinical trials have directly compared kidney-spleen dual regulation with single TCM methods under the same study design. However, current systematic reviews and meta-analyses suggest that TCM, especially when used as an adjunctive therapy, can improve sexual characteristics, hormone levels, and bone-age-related outcomes in girls with precocious puberty (32, 33). A Bayesian network meta-analysis of East Asian traditional medicine interventions for idiopathic CPP further showed that combined treatment modalities, such as herbal medicine combined with GnRHa or external therapy, achieved superior outcomes pertaining to LH, E2, bone-age index, and ovarian volume compared to monotherapies, although the authors also emphasized the need for higher-quality comparative trials (34). These findings indirectly support the clinical rationale of a multi-dimensional and syndrome-differentiated strategy. Future studies should compare dual kidney-spleen regulation with pure yin-nourishing and fire-purging therapy and pure spleen-strengthening and phlegm-resolving therapy using stratified populations, standardized syndrome criteria, endocrine outcomes, metabolic-inflammatory biomarkers, and long-term safety endpoints.

In this review, yin-deficiency fire, phlegm-dampness, and kidney-spleen deficiency are treated as overlapping clinical domains rather than mutually exclusive epidemiological categories. In a questionnaire-based study of 105 girls with precocious puberty and 62 girls with early pubertal development, yin deficiency with fire hyperactivity accompanied by liver depression transforming into fire was the most frequent composite pattern (43.81% and 56.45%, respectively), whereas isolated yin-deficiency fire was uncommon (35). However, as the study was conducted only on female subjects, used fuzzy diagnostic methods, and did not uniformly require biochemical confirmation of CPP, these figures support the prominence of mixed patterns but do not establish prevalence for the proposed three-domain scheme. Accordingly, the choice of TCM prescription followed the dominant pattern – yin-deficiency fire as primary, with liver-fire and phlegm-dampness as common concomitant manifestations. The combinations used in this study, including modified Zhibai Dihuang pills plus Liujunzi decoction and modified Danzhi Xiaoyao powder plus Erchen decoction, remain syndrome-derived options, not independently validated formulation pairs (36). In a Taiwanese cohort of 3495 children with CPP, Zhi-Bai-Di-Huang-Wan constituted 23.73% of the TCM formulas (37). Likewise, in a hospital cohort of 3390 children with idiopathic precocious puberty, it represented 70.62% of 2784 TCM prescriptions and was associated with slower bone maturation and slightly greater predicted height (38). However, these non-randomized data do not establish efficacy or kidney-spleen synergy. In fact, clinical evidence is stronger for kidney-centered yin-nourishing and fire-clearing therapy. For example, in a randomized three-arm study of 106 girls, herbal treatment plus megestrol acetate reduced stimulated LH peak (48.5 ± 37.1 to 12.2 ± 9.3 IU/L), ΔBA/ΔCA (1.35 ± 0.64 to 0.65 ± 0.36), and secondary sexual development, while predicted adult height increased from 153.3 ± 3.6 to 158.5 ± 4.3 cm (39). A case series of 38 female patients reported similar attenuation of skeletal maturation (40), and a later randomized study evaluated ZiYin Xiehuo formula plus megestrol acetate in 61 girls (41). However, concomitant medication and incomplete reporting of trial methods limit the interpretability of these results. The evidence for spleen-strengthening or kidney-spleen dual regulation remains preliminary. A multicenter randomized trial conducted on 143 girls with partial precocious puberty reported short-term differences in uterine volume between the ZiYin Xiehuo and Zishen Qinggan treatment groups, whereas no consistent differences were observed for breast, ovarian, or follicular tissues (42). Furthermore, since the study population did not include children with CPP, the findings cannot be generalized. In a 2025 single-center retrospective study on 62 children with CPP, modified Siwu decoction plus Baizhu ointment reduced the bone-age/chronological-age ratio and some sex steroids, whereas LH and reproductive-organ dimensions did not change significantly. However, the absence of a concurrent control limits causal inference (43). Overall, the available data supports the existence of mixed rather than homogeneous syndromes and provides preliminary evidence for the efficacy of kidney-centered therapy, whereas spleen-related evidence remains limited. A meta-analysis of 17 randomized studies (1590 participants) reported favorable pooled endocrine, organ-volume, and bone-age outcomes for subjects treated with TCM and conventional therapy, although heterogeneity was significant and syndrome-stratified analyses were unavailable (44). Future multicenter trials should enroll subjects with biochemically confirmed CPP, standardize syndrome adjudication, and compare kidney-centered, spleen-centered, dual-regulation, and standard-care strategies in terms of endocrine, growth, metabolic, and safety outcomes.

At the mechanistic level, network-pharmacology and analytical-chemistry studies provide a provisional constituent-target framework for kidney-centered therapy. Analysis of the Zhimu-Huangbai herb pair, which is present in Zhibai Dihuang pills, identified quercetin, kaempferol, and β-sitosterol as key constituents, TP53, JUN, AKT1, ESR1, TNF, IL6, MAPK1, IL1B, and PTGS2 as major nodes, and PI3K-AKT, MAPK, endocrine-resistance, and inflammatory pathways as the significantly enriched pathways (25). Furthermore, analysis of the Fuyou formula by HPLC-MS/MS confirmed the presence of luteolin, quercetin, apigenin, kaempferol, and emodin. In addition, the herb-component-target-pathway network showed significant enrichment of ESR1, IGF1, and the estrogen, MAPK, and PI3K-AKT signaling pathways (45). The Fuyou formula delayed vaginal opening, reduced uterine and ovarian indices in female rats with danazol-induced liver injury, and reduced serum levels of E2, LH, and FSH. At the molecular level, treatment with Fuyou formula downregulated hypothalamic kisspeptin, GPR54, and GnRH, and complementary GT1–7 experiments also showed reduced GPR54/GnRH-PKC/ERK signaling (46). Kangzao granules, which include *Anemarrhena*, *Phellodendron*, *Rehmannia*, and Moutan along with Poria, *Pinellia*, and *Citrus*-derived phlegm-resolving herbs, have shown dual kidney-spleen regulation. Network pharmacology identified 87 bioactive compounds and 110 core proteins, and molecular docking linked the flavonoids to AKT. Furthermore, pharmacodynamic and lipidomic analyses of rats with danazol/high-fat-diet-induced CPP rats showed that treatment with these granules restored hypothalamic fatty acids in a PI3K/AKT-dependent manner (47). Modified Danggui Liuhuang decoction yielded 318 CPP-related targets, and quercetin, kaempferol, and (S)-canadine were connected with ESR1, AKT1, STAT3, TP53, JUN, and hormone-response, JAK-STAT, and HIF-1 pathways. The decoction extract reduced GnRH secretion and neuroendocrine receptor expression in E2-stimulated GT1–7 cells (48). Taken together, these studies suggest that TCM formulations can directly restrain the kisspeptin/GPR54/GnRH–gonadotropin axis and modulate estrogen, PI3K-Akt/MAPK, lipid-metabolic, and inflammatory signaling. Nevertheless, standardized chemical profiling and head-to-head CPP animal studies of modified Zhibai Dihuang pills-Liujunzi decoction or modified Danzhi Xiaoyao powder-Erchen decoction are still required.

## 3 The NEI network theory and its regulatory role in CPP

### 3.1 Composition and crosstalk of the NEI network

**Figure 2:**
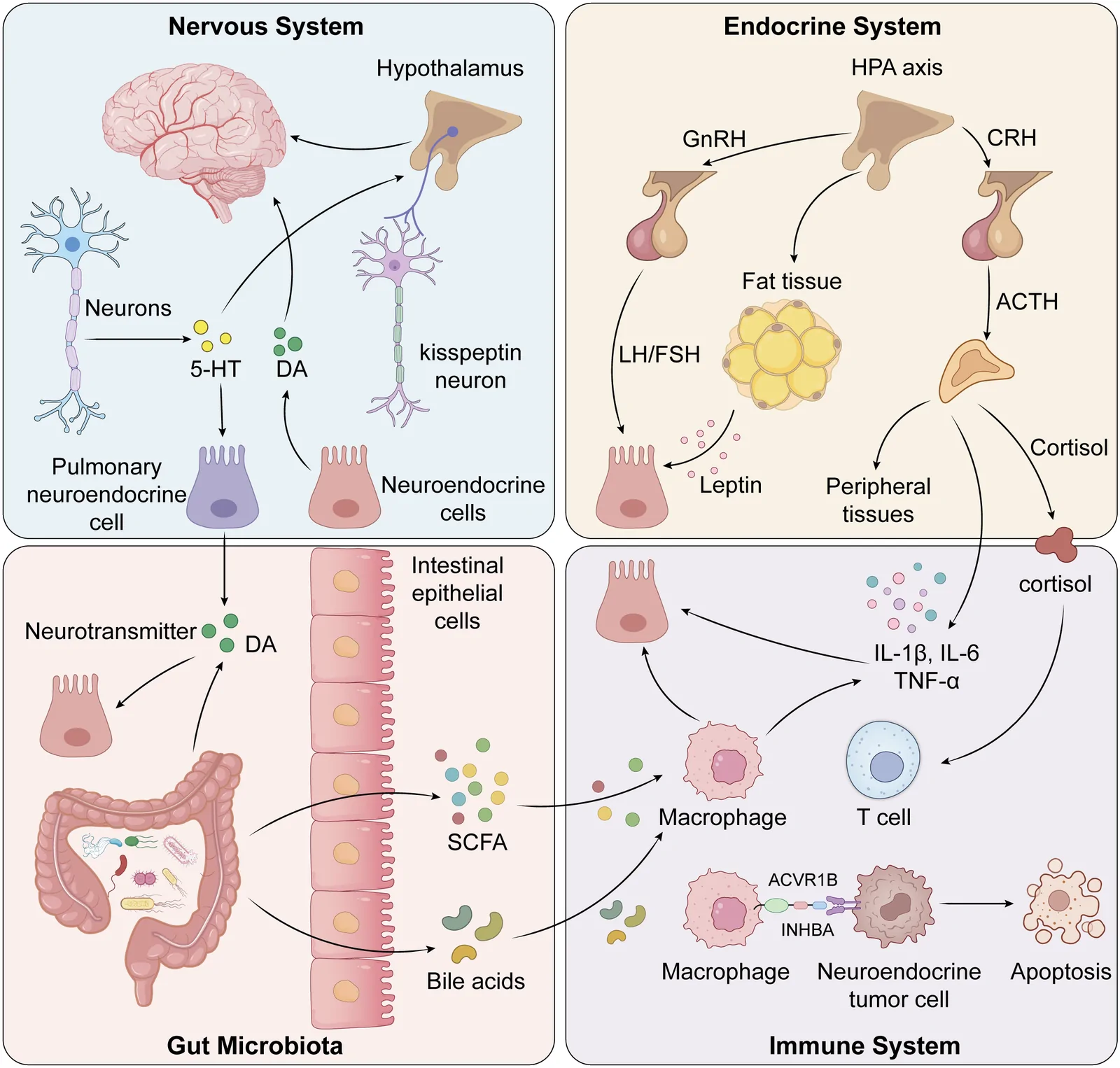
Bidirectional neuro-endocrine-immune signaling and gut microbiota-mediated regulation of the HPG axis. Neural, endocrine, immune, and intestinal compartments exchange neurotransmitters, hormones, cytokines, and microbial metabolites. Hypothalamic kisspeptin/GnRH signaling and pituitary LH/FSH output interact with HPA-axis signaling (CRH-ACTH-cortisol), adipose-derived leptin, and immune mediators. Gut microbiota-derived neurotransmitter signals, SCFAs, and bile acids reach intestinal, immune, and neuroendocrine targets, while macrophage- and T-cell-derived cytokines feed back to the central network. The INHBA-ACVR1B module is shown as an example of paracrine immune-neuroendocrine communication and is not presented as a CPP-specific causal pathway. Solid arrows indicate the direction of secretion, transport, activation, or feedback. 5-HT, 5-hydroxytryptamine; ACTH, adrenocorticotropic hormone; ACVR1B, activin A receptor type 1B; CPP, central precocious puberty; CRH, corticotropin-releasing hormone; DA, dopamine; FSH, follicle-stimulating hormone; GnRH, gonadotropin-releasing hormone; HPA, hypothalamic-pituitary-adrenal; HPG, hypothalamic-pituitary-gonadal; IL, interleukin; INHBA, inhibin subunit beta A; LH, luteinizing hormone; SCFA, short-chain fatty acid; TNF-α, tumor necrosis factor-alpha.

The NEI network is a highly coordinated and bidirectional regulatory system consisting of the nervous, endocrine, and immune systems, which communicate through shared receptors, hormones, neurotransmitters, cytokines, and other signaling molecules to maintain internal homeostasis (49). The HPG axis, which is central to reproductive neuroendocrine regulation, closely interacts with the hypothalamic-pituitary-adrenal (HPA) axis, a key pathway involved in stress and immune responses. When the HPA axis is activated, glucocorticoids can suppress GnRH secretion and reduce pituitary gonadotropin release. In contrast, sex hormones produced through the HPG axis can influence the hypothalamic-pituitary response to corticotropin releasing hormone (CRH), thereby altering the stress response threshold of the HPA axis. This interaction between the HPG and HPA axes reflects the coordinated regulatory nature of the NEI network. The HPA axis also serves as a functional link between neuroendocrine and immune regulation – while HPA activity is influenced by immune signals, HPA-derived hormones can regulate immune cell function (50). Furthermore, the intestinal flora and their metabolites are important regulatory nodes in the NEI network. They connect the gastrointestinal tract with the central nervous system through the gut-brain axis and participate in the control of neuroendocrine activity and immune responses (51). For example, short-chain fatty acids (SCFAs) and bile acids, which are produced through intestinal microbial metabolism, can enter the circulation and act on the central nervous system and immune cells, thereby influencing metabolism, mood, behavior, and immune regulation (52, 53). The involvement of intestinal microbes in HPG axis regulation is illustrated in **Figure 2**.

NEI network imbalance is directly relevant to pediatric endocrine and metabolic conditions, including childhood obesity and obesity-related CPP, metabolic syndrome-associated insulin and leptin resistance during child development, gut dysbiosis in idiopathic CPP, and obesity-associated precocious puberty (26, 54–56). These conditions share several CPP-related mechanisms, such as adipokine disturbance, hypothalamic inflammatory activation, gut microbiota-mediated endocrine signaling, and altered maturation of the HPG axis. The major signaling molecules of the NEI network include neurotransmitters, hormones, and cytokines (49). From the neuroendocrine perspective, CRH, adrenocorticotropic hormone (ACTH), and cortisol regulate the stress response through the HPA axis cascade and also influence immune activity (57). Furthermore, intestinal endocrine cells and some immune cells secrete neurotransmitters such as 5 hydroxytryptamine (5 HT) and dopamine (DA), which contribute to the regulation of intestinal movement, immune cell behavior, and emotional responses (58, 59). Inflammatory cytokines such as interleukin (IL)-1β, tumor necrosis factor α (TNFα), and IL-6 influence hypothalamic function either by crossing the blood-brain barrier or by transmitting signals through the vagus nerve. They can also act directly on neurons and affect neural plasticity, behavior, and neuroendocrine activity (60). Leptin, an adipocyte-derived hormone-like cytokine, plays an important role in appetite control and energy balance by linking nutritional and metabolic status with NEI regulation (53). Altogether, these molecules form multiple feedback and feed-forward loops within the NEI network. For example, stress induced HPA axis activation leads to cortisol release, which then suppresses excessive production of pro-inflammatory cytokines through receptor-mediated mechanisms, thereby creating a negative feedback pathway that helps control overactive inflammatory responses (50).

Kisspeptin-producing neurons are located in the hypothalamus and play an essential role in pubertal development and reproductive function by regulating HPG axis activation (61). Studies show that signal transduction and differentiation of pulmonary neuroendocrine cells drive several pulmonary phenotypes, suggesting significant regulatory roles of these cells within specific tissue microenvironments (61). Although there is limited direct evidence of the role of kisspeptin in the NEI network, the current consensus is that neuroendocrine cells receive signals from classical neurotransmitters and hormones, while also sensing metabolic, stress-related, and immune signals (57). For example, chronic psychological stress can activate both the sympathetic nervous system and the HPA axis, leading to broad transcriptional changes that regulate circadian rhythm, immune response, and lipid metabolism in local tissues. This underscores the central role of the neuroendocrine system in connecting stress signals with immune and metabolic homeostasis (57). A similar communication pattern is also observed in the tumor microenvironment, where neuroendocrine tumor cells interact with immune cells. For instance, tumor associated macrophages can communicate with neuroendocrine tumor cells through the INHBA and ACVR1B receptors and regulate their apoptosis. This further supports the role of neuroendocrine components as communication hubs between the nervous and immune systems (62). In summary, kisspeptin neurons and other neuroendocrine cells are positioned at important crossing points of the NEI network, where they convert metabolic, immune, stress-related, and hormonal information into integrated neuroendocrine responses, thereby maintaining systemic homeostasis.

### 3.2 NEI network imbalance and the pathogenesis of CPP

**Figure 3:**
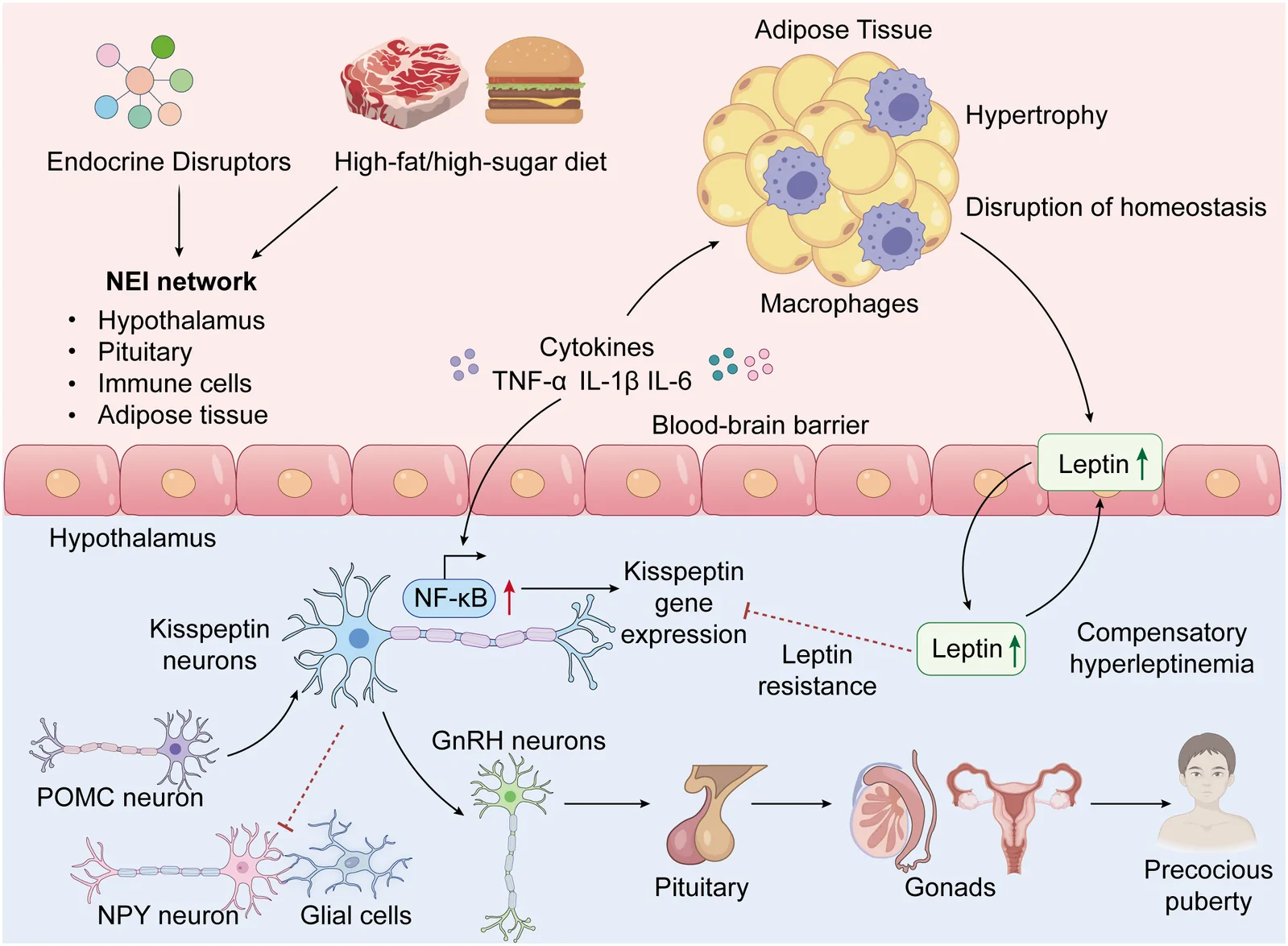
Adipose tissue-immune-neuroendocrine crosstalk promoting premature activation of the HPG axis. Endocrine disruptors and a high-fat/high-sugar diet perturb the NEI network and favor adipocyte hypertrophy and macrophage infiltration. Adipose-derived leptin and the cytokines TNF-α, IL-1β, and IL-6 reach the hypothalamus through circulating or blood-brain barrier-associated signaling. Increased NF-κB activity, glial activation, altered POMC/NPY input, and increased kisspeptin expression then enhance GnRH neuronal output, followed sequentially by pituitary and gonadal activation and precocious puberty. Leptin resistance and compensatory hyperleptinemia reinforce this metabolic-inflammatory loop. Solid arrows indicate downstream activation or signal transfer; dashed red lines indicate disrupted or pathological regulatory relationships; upward arrows indicate increased abundance or activity. CPP, central precocious puberty; GnRH, gonadotropin-releasing hormone; HPG, hypothalamic-pituitary-gonadal; IL, interleukin; NEI, neuro-endocrine-immune; NF-κB, nuclear factor kappa B; NPY, neuropeptide Y; POMC, proopiomelanocortin; TNF-α, tumor necrosis factor-alpha.

The development of CPP is the result of the combined influence of neuroendocrine, metabolic, immune, inflammatory, genetic, and environmental mechanisms. In recent years, the role of the nervous system in CPP has garnered significant attention since the early activation of the HPG axis is closely controlled by central neuroendocrine signals. The mechanism by which adipose tissues, immune system, and the neuroendocrine system contribute to early HPG axis activation is shown in **Figure 3**. Any imbalance in the NEI network – caused by excessive nutrition and environmental endocrine disruptors among other factors – can promote the premature activation of the HPG axis. Long-term energy excess, especially from diets rich in fat and carbohydrates, increases the risk of childhood overweight and obesity. At the same time, it acts as a chronic metabolic stressor that may directly interfere with NEI signal transmission. Furthermore, environmental endocrine disruptors, such as bisphenol A and phthalates, exhibit hormone-like or hormone-blocking effects that can disturb normal endocrine activity in the hypothalamus, pituitary gland, and related reproductive tissues. These metabolic and environmental factors can directly or indirectly influence the activity and expression of kisspeptin-producing neurons, especially in hypothalamic regions such as the arcuate nucleus. Kisspeptin is one of the strongest stimulators of GnRH pulse generation, and its upregulation is a biological trigger of puberty. Therefore, disruption of the NEI network can lead to the premature disinhibition of the kisspeptin system, resulting in overactivation of kisspeptin neurons. These hyperactive neurons then stimulate the GnRH pulse generator, and shift GnRH secretion into an early high frequency and high amplitude pattern, eventually leading to early gonadal activation and CPP. Thus, the development of CPP is a cascade process wherein environmental and metabolic stressors disturb the NEI network and the neuroendocrine center, thereby initiating premature pubertal development.

Chronic low-grade inflammation is another major sign of immune imbalance within the NEI network and plays a pathological role in obesity-related CPP. Adipose tissue is not only a storage site for excess energy but also an active endocrine and immune organ. The excessive growth and enlargement of adipose tissues can cause local hypoxia, cellular stress, and immune activation, which promote the infiltration of macrophages and other immune cells. The activated immune cells release pro-inflammatory cytokines, including TNFα, IL-1β, and IL-6. Continuous release of these cytokines induces a systemic state of chronic low-grade inflammation. Furthermore, these cytokines can cross the blood-brain barrier or interact with cerebrovascular endothelial cells and target neuronal populations and glial cells in the hypothalamic region. Specifically, pro-inflammatory cytokines may impair the functions of the POMC and NPY neurons, which are involved in energy balance and reproductive control. Furthermore, inflammatory signals can alter kisspeptin gene expression in neurons or modify neuronal excitability through the NF-κB signaling pathway, which may weaken the normal inhibitory control of GnRH pulse generator or increase stimulatory input to GnRH neurons. Therefore, the inflammatory environment acts as a bridge between peripheral metabolic disturbance and central reproductive endocrine control, suggesting that NEI network imbalance may contribute to the development of CPP through immune-inflammatory mechanisms.

**Figure 4:**
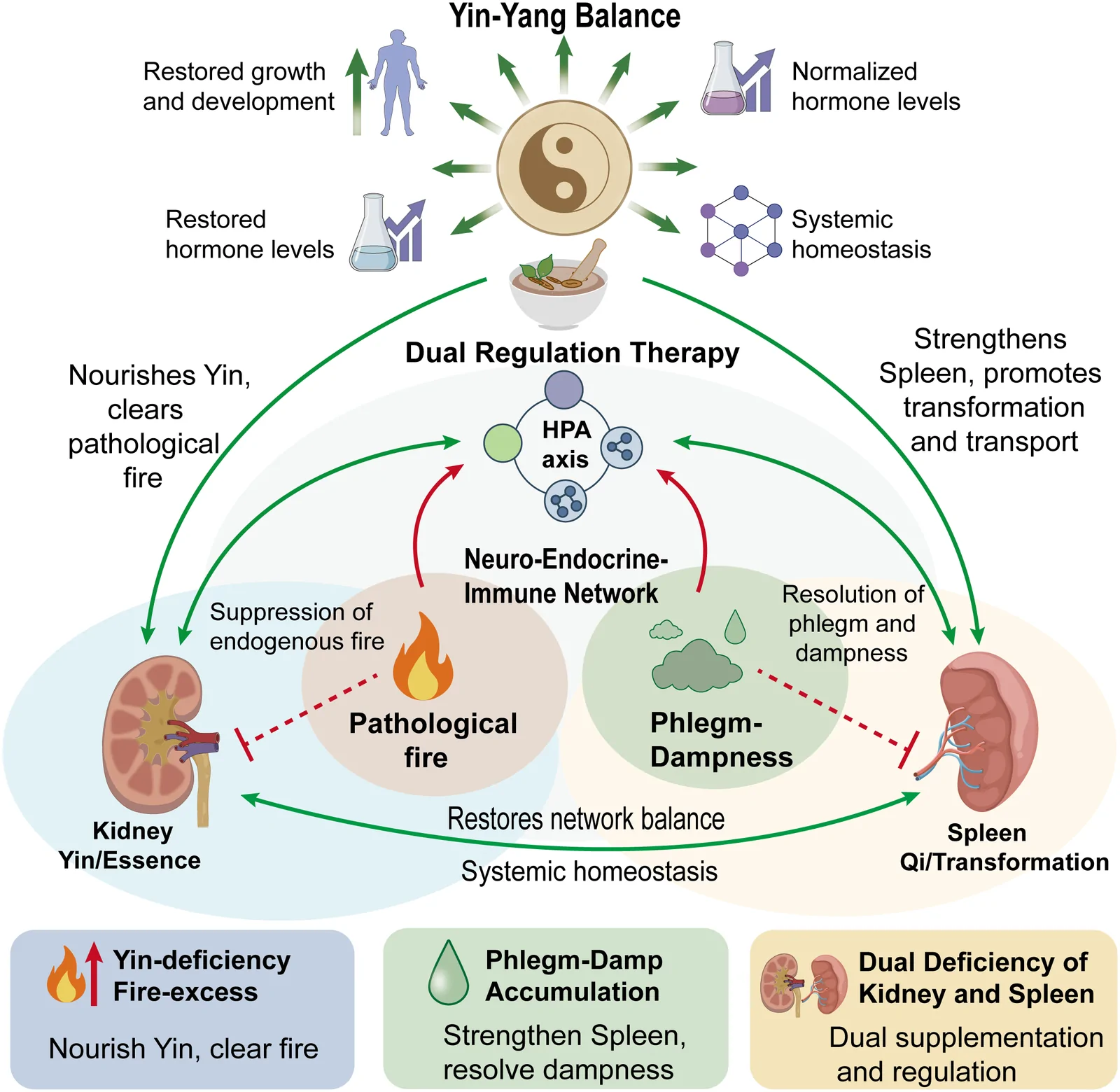
Proposed kidney-spleen dual-regulation framework for restoring neuro-endocrine-immune homeostasis in pediatric CPP. The lower panels represent three overlapping syndrome domains: kidney-yin deficiency with fire excess, phlegm-damp accumulation due to impaired spleen transformation, and combined kidney-spleen deficiency. Kidney-yin nourishment and fire clearance suppress pathological fire, whereas spleen-strengthening and phlegm resolution reduce dampness and improve transportation and transformation. Coordinated intervention may attenuate impairment to the HPA axis and wider NEI network, restore kidney-spleen reciprocity, normalize endocrine signaling, and support growth and systemic homeostasis. Green arrows denote physiological or therapeutic restoration, red arrows denote pathological excitation, dashed red blunt-ended lines denote pathological impairment, and outward arrows indicate downstream recovery. Kidney and spleen are used here as TCM functional constructs rather than direct anatomical equivalents. CPP, central precocious puberty; HPA, hypothalamic-pituitary-adrenal; NEI, neuro-endocrine-immune; TCM, traditional Chinese medicine.

Leptin is another key molecule that links adipose tissue, metabolism, immunity, and reproductive endocrinology within the NEI network. It plays an important role in obesity-related CPP and reflects the close interaction between endocrine and immune signaling. Leptin is mainly secreted by white adipocytes, and its level increases with body fat accumulation. Under normal physiological conditions, leptin signals the central nervous system that the body has sufficient energy stores, and acts as a permissive signal for the initiation of puberty. In addition, leptin indirectly promotes kisspeptin and GnRH release through leptin receptor-related pathways in the hypothalamus. Obesity-associated CPP is often accompanied by hyperleptinemia and leptin resistance. High leptin levels not only overstimulate the hypothalamic reproductive center but can also amplify chronic low-grade inflammation by activating monocytes and macrophages and promoting differentiation of specific T cell subsets along with TNFα and IL-6. This inflammatory response may further worsen central and peripheral leptin resistance, creating a self-reinforcing cycle, which lowers the responsiveness of the arcuate nucleus to normal leptin signaling. To compensate for this reduced response, the body produces more leptin, which then stimulates the hypothalamic reproductive axis likely through kisspeptin regulation and early GnRH secretion. Therefore, leptin should be viewed not only as an endocrine hormone but also as an immune-related cytokine-like mediator. It serves as an important hub in NEI network imbalance and contributes to the dynamic process of premature sexual maturation. In addition to adipose tissue-derived immune activation, adaptive immune dysfunction may also participate in CPP pathogenesis. Imbalance in T lymphocyte subsets, including Th1, Th2, and Treg cells, along with increased infiltration of pro-inflammatory macrophages in peripheral immune organs, may weaken immune tolerance. These changes can further activate NF-κB and NLRP3 inflammatory signaling pathways, resulting in increased hypothalamic neuroinflammation that can directly support premature HPG axis activation and contribute to the progression of CPP. The integrated mechanisms by which dual regulation of the kidney and spleen may intervene in this network are illustrated in **Figure 4**.

## 4 Kidney-spleen dual regulation along the hypothalamic–pituitary–gonadal axis

**Figure 5:**
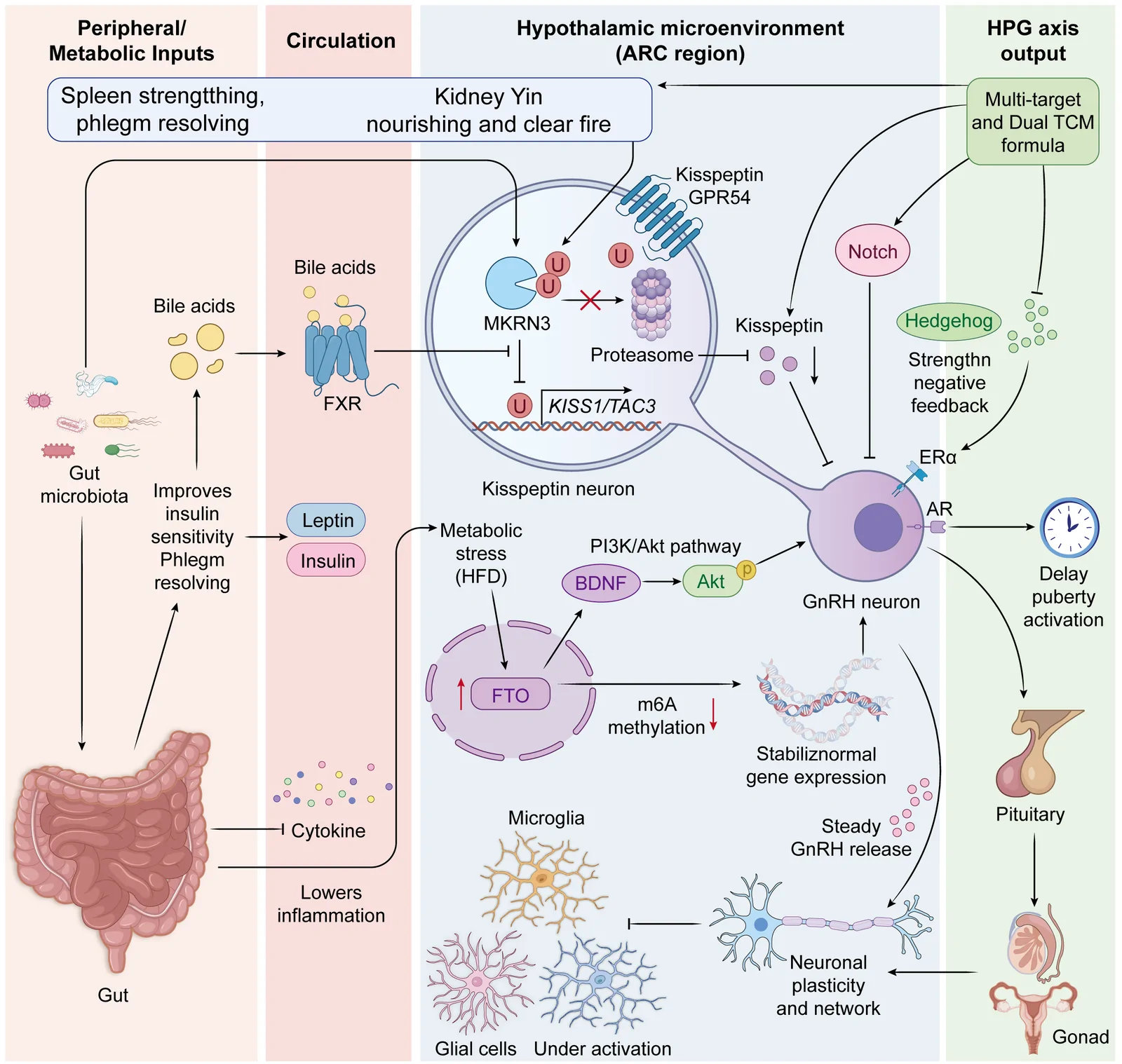
Proposed multi-pathway mechanisms of kidney-spleen dual-regulating therapy in CPP. Peripheral and central pathways are arranged from left to right. Spleen-strengthening and phlegm-resolving therapy reshapes the gut microbiota and bile acid/FXR signaling, improves insulin sensitivity, normalizes leptin and insulin signals, and reduces circulating cytokine input. Kidney-yin nourishment and fire clearance can restrain the hypothalamic kisspeptin-GPR54-GnRH pathway and neuroglial activation. MKRN3-mediated ubiquitin-dependent repression of KISS1/TAC3 and GnRH-related transcription represents an upstream prepubertal brake; conversely, the FTO-BDNF-PI3K/Akt branch links reduced m6A methylation with increased GnRH drive. Steroid-receptor, Notch, and Hedgehog signaling are shown as candidate feedback pathways. Solid arrows indicate activation, transport, or downstream progression, blunt-ended lines indicate inhibition, red upward or downward arrows indicate increases or decreases respectively. Direct effects of the representative formulas on MKRN3 or FTO have not been established. Akt, protein kinase B; AR, androgen receptor; ARC, arcuate nucleus; BDNF, brain-derived neurotrophic factor; CPP, central precocious puberty; ERα, estrogen receptor-alpha; FTO, FTO alpha-ketoglutarate-dependent dioxygenase; FXR, farnesoid X receptor; GnRH, gonadotropin-releasing hormone; GPR54, G protein-coupled receptor 54 (KISS1 receptor); HFD, high-fat diet; HPG, hypothalamic-pituitary-gonadal; KISS1, kisspeptin 1 gene; m6A, N6-methyladenosine; MKRN3, makorin ring finger protein 3; PI3K, phosphoinositide 3-kinase; TAC3, tachykinin precursor 3; TCM, traditional Chinese medicine; U, ubiquitin.

**Figure 6:**
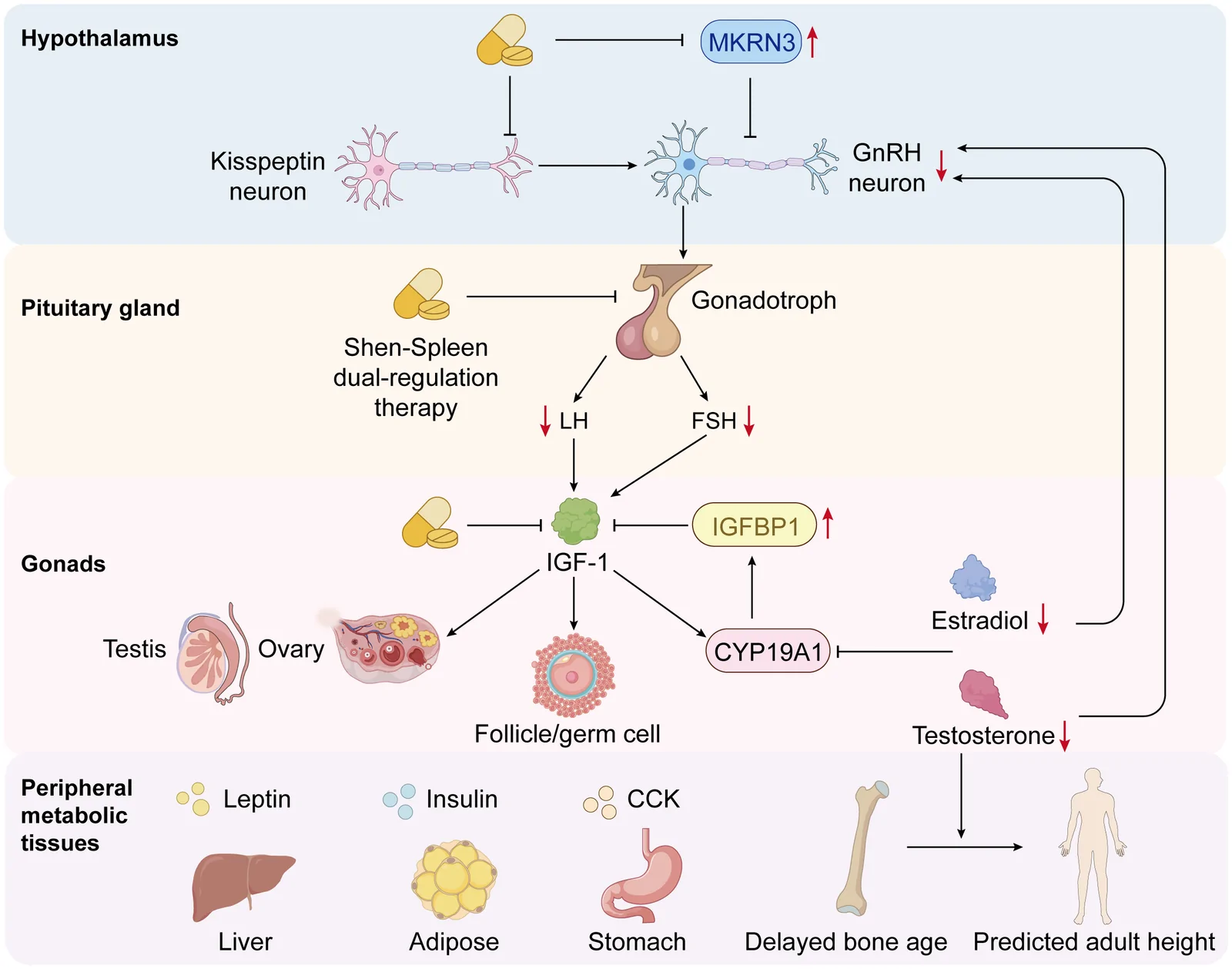
Anatomically ordered model of kidney-spleen dual-regulating therapy along the HPG axis in CPP. At the hypothalamic level, kisspeptin activates GnRH neurons, whereas MKRN3 acts as an upstream inhibitory brake. GnRH stimulates pituitary gonadotrophs and downstream LH/FSH release; gonadotropin and IGF-1 signaling then support follicular or germ-cell maturation, aromatase-dependent steroidogenesis, and estradiol or testosterone production. IGFBP1 limits IGF-1 bioavailability, and sex steroids provide feedback to the hypothalamus and pituitary. Peripheral leptin, insulin, and CCK provide metabolic input. Kidney-spleen dual-regulating therapy (labeled Shen-Spleen in the panel) can reduce kisspeptin/GnRH drive and downstream gonadotropin and sex-steroid exposure, thereby slowing bone-age advancement and preserving adult-height potential. Solid arrows indicate activation or downstream progression, blunt-ended lines indicate inhibition, reverse arrows indicate endocrine feedback, and red upward or downward arrows indicate increases or decreases. MKRN3, IGF-1/IGFBP1, and CYP19A1 are the candidate nodes; direct modulation by the representative formulas remains to be demonstrated. CCK, cholecystokinin; CPP, central precocious puberty; CYP19A1, cytochrome P450 family 19 subfamily A member 1 (aromatase); FSH, follicle-stimulating hormone; GnRH, gonadotropin-releasing hormone; HPG, hypothalamic-pituitary-gonadal; IGF-1, insulin-like growth factor 1; IGFBP1, insulin-like growth factor-binding protein 1; LH, luteinizing hormone; MKRN3, makorin ring finger protein 3.

In this section, we have discussed the anatomical and functional effects of kidney-spleen dual regulation. The hypothalamic kisspeptin/GPR54/GnRH system is the principal mechanistic node, followed by pituitary gonadotroph responsiveness, gonadal steroidogenesis, and IGF-1-associated signaling as downstream or auxiliary levels. This hierarchy separates candidate molecular mechanisms from clinical endocrine outcomes and clarifies the multilevel model summarized in **Figures 5**, **6**.

### 4.1 Hypothalamic Kiss1/GPR54/GnRH signaling as the primary target

The hypothalamus is the initiating node of the HPG axis. At pubertal onset, kisspeptin binds GPR54 (KISS1R) on GnRH neurons and increases pulsatile GnRH secretion. MKRN3, an upstream inhibitor, is maternally imprinted and paternally expressed, and high prepubertal hypothalamic expression (Mkrn3 in rodents) declines before normal puberty. A loss-of-function variant of Mkrn3 on the expressed paternal allele can prematurely release the restraint on GnRH secretion (5, 6). MKRN3 represses KISS1 and TAC3 promoter activity, and also ubiquitinates MBD3, thus limiting TET2-dependent demethylation of the GNRH1 promoter and maintaining GNRH1 repression (6, 63). Thus, reduced MKRN3 expression or function is permissive for pubertal onset, whereas increased functional MKRN3 would reinforce prepubertal restraint. FTO represents a distinct epitranscriptomic mechanism of GnRH regulation. In female rodents, puberty is accompanied by an increase in hypothalamic FTO expression and decline in the levels of methylated Bdnf mRNA. FTO overexpression in the arcuate nucleus of female rats reduced Bdnf mRNA-m6A levels, increased BDNF/PI3K/Akt signaling and GnRH expression, and hastened vaginal opening, while FTO deficiency resulted in the opposite phenotype (10). The researchers also recorded higher circulating BDNF in girls with CPP but did not measure hypothalamic FTO. Therefore, FTO/BDNF was identified as a regulator of pubertal timing in a preclinical model, but not a confirmed biomarker of human CPP or direct target of TCM.

Various TCM formulations have been shown to influence the downstream phenotypes of these gene-level mechanisms. For instance, the Fuyou formula delayed vaginal opening, reduced serum LH, FSH, and estradiol levels, and downregulated hypothalamic kisspeptin, GPR54, and GnRH in female rats with danazol-induced liver fibrosis, and inhibited GPR54/GnRH-PKC/ERK signaling in GT1–7 cells (46). Likewise, Kangzao granules corrected hypothalamic lipid disturbances in a high-fat-diet-associated CPP model, and network pharmacology and lipidomics identified the involvement of PI3K/Akt signaling (47). However, neither study assessed MKRN3 expression or function, parental imprinting, FTO abundance, or Bdnf m6A status. Thus, attenuation of kisspeptin/GPR54/GnRH or PI3K/Akt signaling does not establish direct modulation of MKRN3 or FTO. This, the kisspeptin/GPR54/GnRH cascade is the more direct target, whereas the involvement of MKRN3 or FTO remains exploratory at present. Spleen-regulating formulations may reinforce the effect on hypothalamic signaling by reducing peripheral inputs that sensitize the hypothalamus. Hyperleptinemia, insulin resistance, high-fat-diet-induced inflammation, and microbiota-derived metabolites can alter kisspeptin neuronal activity and the local glial environment (27, 64–67). Spleen-invigorating and phlegm-resolving herbs may therefore act indirectly by improving metabolic signaling, reshaping microbial and bile acid profiles, and limiting microglial activation, whereas kidney yin-nourishing and ministerial fire-clearing herbs may act more proximally on hypothalamic neuroendocrine signaling. The proposed benefit of dual regulation is thus a coordinated reduction in both intrinsic GnRH-neuronal excitation and peripheral metabolic-inflammatory pressure.

### 4.2 Pituitary gonadotroph responsiveness and gonadotropin secretion

Pulsatile GnRH released into the hypophyseal portal circulation activates pituitary gonadotrophs, thereby promoting the synthesis and secretion of LH and FSH. Increased LH secretion, particularly the peak LH response during a GnRH stimulation test, is a principal biochemical marker of central HPG-axis activation (68). Clinical studies have shown that Chinese herbal interventions may reduce basal or stimulated LH and FSH and improve bone-age-related outcomes in some children with CPP (25, 32–34). These changes are compatible with diminished GnRH drive and may also reflect altered gonadotroph responsiveness. However, the available cellular evidence is centered largely on hypothalamic GnRH neuronal models (46, 48), and does not establish a direct effect on pituitary GnRH-receptor abundance, gonadotropin-subunit transcription, or receptor desensitization. Consequently, reduced LH and FSH should be interpreted primarily as downstream endocrine evidence of attenuated HPG-axis activity, while a direct action of kidney-spleen prescriptions on the pituitary hormones remains to be verified. Therefore, this therapeutic strategy should not be regarded as pharmacologically equivalent to a GnRH agonist.

### 4.3 Gonadal steroidogenesis and sex-steroid feedback

At the gonadal level, LH and FSH stimulate ovarian or testicular maturation and the production of estradiol or testosterone. A reduction in gonadotropin drive can therefore lower sex-steroid exposure, slow secondary sexual development, and mitigate accelerated skeletal maturation (2, 69). The reported decrease in estradiol or testosterone levels with dual kidney-spleen regulation can be interpreted within this downstream sequence: hypothalamic restraint reduces pituitary LH/FSH secretion, which subsequently limits gonadal steroidogenesis. A supplementary, tissue-local action is possible but less firmly supported. Network analyses of CPP formulas have identified hormone-response nodes such as ESR1, AKT1, MAPK-related proteins, and steroidogenic pathways (45, 48), suggesting potential effects on sex-steroid receptor signaling or enzymes such as CYP19A1. The gonadal microenvironment also responds to cytokines and growth factors that can influence steroid production independently of circulating gonadotropin levels (70). Nevertheless, no study has demonstrated the direct regulatory effect of kidney-spleen formulations on gonadal steroidogenic enzymes in the context CPP.

### 4.4 IGF-1-associated growth and reproductive signaling

IGF-1-associated signaling is best considered a secondary interface between nutritional status, somatic growth, and reproductive maturation rather than part of the core GnRH-LH/FSH cascade. Experimental evidence indicates that IGF-1 can facilitate follicular development and steroidogenic competence, whereas IGF-binding proteins, including IGFBP-1, reduce IGF-1 bioavailability and may restrain reproductive activity across hypothalamic, pituitary, and gonadal sites (71). These observations provide biological plausibility for the contribution of IGF-1 to pubertal progression, although much of the supporting evidence is derived from non-CPP models. Modern pharmacological studies provide preliminary evidence that TCM formulations affect this pathway. Integrated analysis of the Fuyou formula identified IGF1 and ESR1 as network targets (45), while subsequent studies showed reduced IGF-1 and IGF-1R expression, along with inhibition of Kiss1/GPR54/GnRH signaling in hypothalamic GnRH neurons (72). These findings suggest that IGF-1 signaling may amplify central neuroendocrine sensitivity or peripheral gonadal maturation, and that its modulation could complement direct restraint of the GnRH pulse generator. These findings do not, however, establish systemic or gonadal IGF-1 regulation by the representative kidney-spleen prescriptions described in subsection 2.2. Future mechanistic studies should distinguish circulating GH/IGF-1 effects from tissue-specific IGF-1/IGF-1R signaling, and evaluate these measures alongside LH, FSH, estradiol or testosterone, steroidogenic enzymes, and bone-age progression. Taken together, hypothalamic inhibition of Kiss1/GPR54/GnRH is the primary mechanism, followed by reduced pituitary gonadotropin output and gonadal steroidogenesis, with IGF-1-associated signaling as an additional regulator of growth and reproduction.

## 5 Effects of kidney-spleen dual regulation on immune homeostasis and inflammatory networks

### 5.1 Regulation of immune cell function and cytokine profile

**Figure 7:**
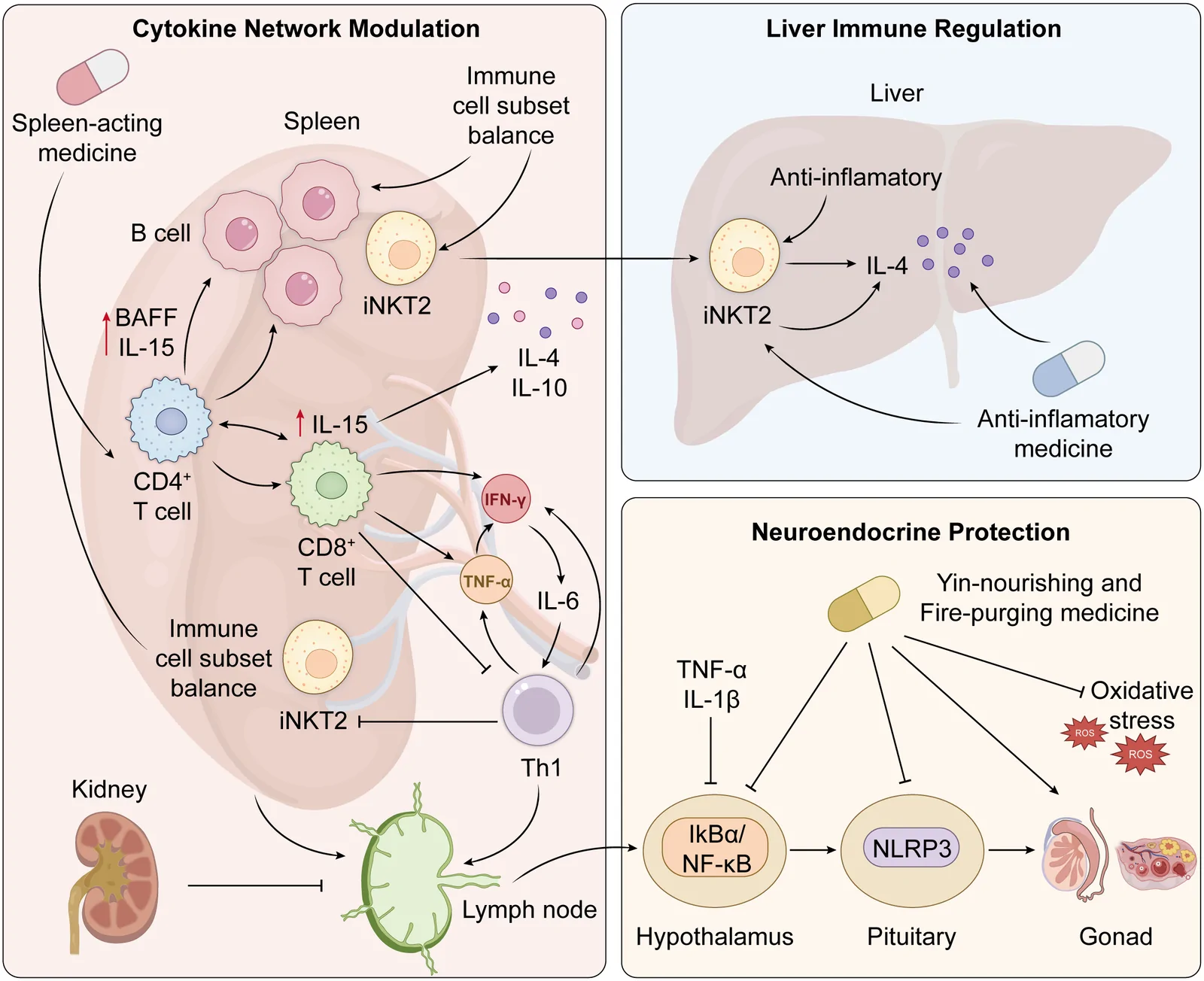
Proposed immune-network modulation and neuroendocrine protection by kidney-spleen dual-regulating therapy. Spleen-acting therapy is proposed to rebalance splenic B cells, CD4+ T cells, CD8+ T cells, Th1 cells, and iNKT2 cells, as well as BAFF/IL-15, IL-4/IL-10, and IFN-γ/TNF-α/IL-6 signaling. iNKT2 trafficking to the liver and IL-4 production represent anti-inflammatory cross-organ regulation. In parallel, yin-nourishing and fire-purging therapy may reduce TNF-α/IL-1β signaling, IκBα/NF-κB activation, NLRP3 inflammasome activity, and oxidative stress across hypothalamic, pituitary, and gonadal tissues. Solid arrows indicate activation, secretion, trafficking, or downstream propagation, blunt-ended lines indicate inhibition, red upward arrows indicate increased mediator abundance. These mechanisms are hypotheses at present, and CPP-specific validation remains limited. BAFF, B-cell-activating factor; CD, cluster of differentiation; CPP, central precocious puberty; IFN-γ, interferon-gamma; IκBα, inhibitor of nuclear factor kappa B-alpha; IL, interleukin; iNKT2, type 2 invariant natural killer T cell; NF-κB, nuclear factor kappa B; NLRP3, NOD-, LRR- and pyrin domain-containing protein 3; ROS, reactive oxygen species; Th1, type 1 helper T cell; TNF-α, tumor necrosis factor-alpha.

The spleen is a major immune organ, and its function determines the overall immune status. In TCM, spleen deficiency is commonly related to weakened or disordered immune function. Modern research has also established the immunoregulatory effect of spleen-invigorating Chinese medicinal herbs. The mechanisms through which dual regulation of the kidney and spleen modulates the immune microenvironment and protects neuroendocrine function are illustrated in **Figure 7**. Studies on animal models have shown that the spleen is an important site for the activation of several immune cells, including T cells, B cells, natural killer T cells, and the production of cytokines such as BAFF and IL-15 (73, 74). Impaired splenic function, such as congenital asplenia, can disturb immune homeostasis and trigger excessive systemic inflammation, which is characterized by increased production of pro-inflammatory cytokines, including TNFα and IFNγ, and the suppression of anti-inflammatory cytokines (75). These findings suggest that spleen-invigorating therapy may help treat spleen deficiency syndrome by supporting immune homeostasis rather than by acting on a single immune organ. Furthermore, this effect is likely mediated by restoring the balance of immune cell subsets, especially CD4+ and CD8+ T cells, and through regulation of the cytokine network (76, 77). Therefore, spleen-invigorating herbs may improve the systemic immune microenvironment by acting on the spleen and related human lymphoid tissues, including lymph nodes and bone marrow (78), and influence regulatory T-cell activity, macrophage polarization, and NLRP3-related inflammatory signaling, although direct evidence in pediatric CPP remains limited. These immune pathways should therefore be treated as candidate mechanisms requiring CPP-specific validation rather than established clinical effects.

Previous studies have indicated that children with CPP may show Th1/Th2 cytokine imbalance or chronic low-grade inflammation. The Th1 immune response is mainly driven by pro-inflammatory cytokines such as IFNγ and TNFα, whereas the Th2 response is mainly associated with anti-inflammatory cytokines such as IL-4 and IL-10 (74). T-bet is a transcription factor that promotes Th1 cell differentiation and IFNγ secretion (79), and its action may be disrupted under pathological conditions. For example, pathogenic infection under immunosuppressive conditions may produce a mixed Th1/Th2 cytokine response consisting of IFNγ, TNFα, IL-2, and IL-4, which is closely related to disease severity (80). Therefore, dual regulation of the kidney and spleen may contribute to CPP treatment by helping restore the balance between Th1 and Th2 immune responses. Some immunoregulatory interventions have been shown to reduce inflammatory mediators, including IL-6 and TNFα (81). In addition, adoptive transfer of anti-inflammatory immune cell subsets, such as iNKT2 cells that mainly secrete IL-4, has shown good biosafety. Furthermore, these cells mainly localize in the liver and spleen, suggesting a role in the formation of an anti-inflammatory immune microenvironment (82). Based on these findings, simultaneous regulation of the kidney and spleen may help correct the inflammatory tendency in children with CPP by down regulating pro-inflammatory factors and upregulating anti-inflammatory mediators (83).

Most TCM herbs used for CPP treatment belong to Yin-nourishing and fire-purging medicines, which are applied to clear internal deficient fire. Modern pharmacological evidence suggests that these medicines have anti-inflammatory and antioxidant effects, which may reduce inflammatory injury in neuroendocrine tissues. Persistent inflammation and oxidative stress can damage neuroendocrine centers, especially the hypothalamus, and disturb the normal pulsatile secretion and release of GnRH. Low-grade inflammation has also been associated with tissue injury and immune dysfunction in many disease processes (84). Studies show that activation of the IκBα/NF-κB signaling pathway and NLRP3-mediated pyroptosis are important mechanisms involved in inflammatory tissue damage (81). In addition, cytokine-mediated inflammation may contribute to organ damage in different pathological conditions, thereby warranting anti-inflammatory therapeutic strategies (85). For example, in kidney transplant models, regulation of T cell function can help control the inflammatory microenvironment within the graft (77). Yin-nourishing and fire-purging medicines may reduce inflammation in tissues related to the HPG axis by blocking pro-inflammatory pathways and protect neuroendocrine tissues from inflammatory mediators such as TNFα and IL-1β. This inference is strongest when supported by CPP or hypothalamic-inflammation models and should be distinguished from evidence derived from unrelated inflammatory diseases. These formulations may also reduce oxidative stress and create a more conducive environment for the recovery of NEI network function, which may represent one of the key mechanisms of CPP treatment (86).

### 5.2 Effects on adipokines and metabolic inflammation

**Figure 8:**
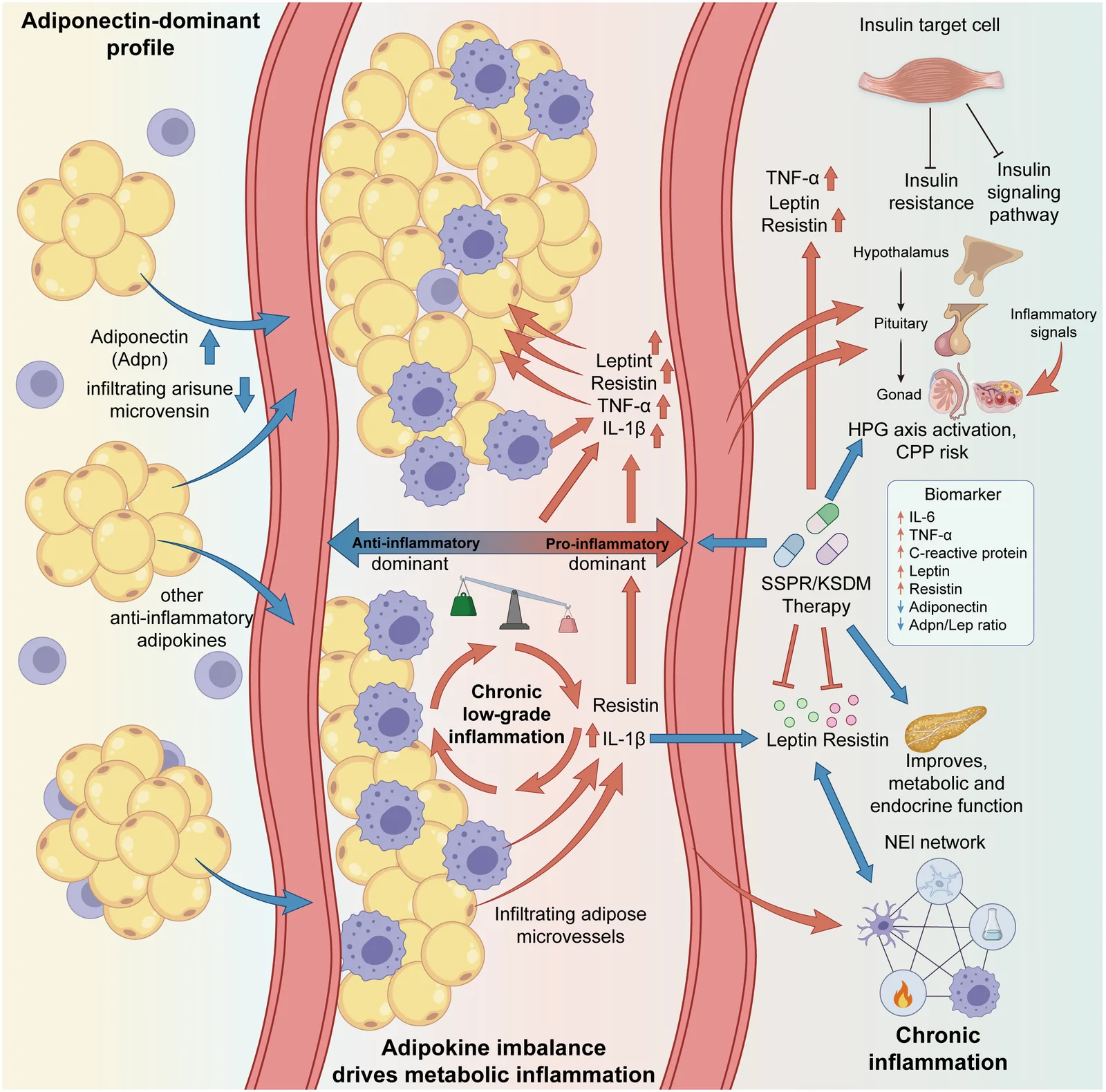
Proposed correction of adipokine imbalance and metabolic inflammation by kidney-spleen dual-regulating therapy. In an adiponectin-dominant state, adiponectin and other anti-inflammatory adipokines limit immune-cell infiltration and preserve insulin sensitivity. Adipocyte hypertrophy and leukocyte infiltration shift adipose tissue toward a pro-inflammatory profile with increased leptin, resistin, TNF-α, and IL-1β production, thereby sustaining chronic low-grade inflammation. Circulating adipokines and cytokines impair insulin signaling and transmit inflammatory and metabolic input to the hypothalamus, pituitary, and gonads, which increases HPG-axis activation and CPP risk. SSPR/KSDM therapy can lower leptin, resistin, and inflammatory biomarkers, increase adiponectin levels, improve the adiponectin/leptin ratio (LEP/ADPN ratio), and stabilize the NEI network. Blue arrows indicate protective or therapeutic effects, red arrows indicate pathological propagation, blunt-ended lines indicate inhibition, and upward or downward arrows indicate increases or decreases. ADPN, adiponectin; CPP, central precocious puberty; HPG, hypothalamic-pituitary-gonadal; IL, interleukin; KSDM, kidney-spleen dual modulation; LEP, leptin; NEI, neuro-endocrine-immune; SSPR, spleen-strengthening and phlegm-resolving; TNF-α, tumor necrosis factor-alpha.

Splenic hypofunction is the basis of phlegm-dampness constitution and obesity in TCM. Therefore, spleen invigoration and phlegm resolution may improve these metabolic abnormalities. The mechanistic basis of kidney-spleen dual regulation therapy is correcting adipokine disorder and metabolic inflammation, which stabilizes the NEI network (**Figure 8**). In obese individuals, the adipose tissue does not simply store energy; rather, it functions as an active endocrine organ. Furthermore, the secretory pattern of dysfunctional adipose tissue changes from an anti-inflammatory to a pro-inflammatory state. This shift promotes chronic low-grade inflammation, also known as metabolic inflammation, which is closely related to insulin resistance and metabolic disorders (87). The adipocytes and infiltrating leukocytes in obese individuals release excessive pro-inflammatory adipokines and cytokines, including leptin, resistin, TNFα, and IL-1β, while the secretion of protective anti-inflammatory adipokines such as adiponectin is reduced (87, 88).

This adipokine imbalance plays a direct role in the initiation and persistence of metabolic inflammation (87). Previous studies have shown that the Mediterranean diet combined with physical activity reduced body weight and BMI within two years in metabolically healthy prepubertal obese children, which was accompanied by the reduction in inflammatory markers such as IL-6, TNFα, and C reactive protein. However, the adipokine profile, including reduced adiponectin and increased resistin, did not show a similar improvement, suggesting that adipokine disturbance is strongly linked with obesity. Similarly, patients with metabolic syndrome and low vitamin D levels show significantly increased serum leptin, resistin, and TNFα levels, along with decreased adiponectin levels, which confirms the co-existence of adipokine disorder and inflammation (88). Therefore, the therapeutic value of invigorating the spleen and resolving phlegm in individuals with splenic hypofunction and internal phlegm-dampness may lie in its ability to correct abnormal adipokine secretion. In particular, it may reduce elevated leptin and resistin levels and mitigate chronic inflammation induced by the adipose tissue, thus providing a promising approach for the management of obesity and obesity-related disorders. Furthermore, this therapy may reshape the immune microenvironment of the adipose tissue by reducing the infiltration of pro-inflammatory immune cells, such as macrophages and neutrophils, and by suppressing the secretion of TNFα, IL-1β, and IL-6, and therefore interrupt the immune metabolic inflammatory cycle that contributes to CPP progression.

The simultaneous regulation of the kidney and spleen may also indirectly influence the NEI axis by restoring adipokine homeostasis and increasing the levels of adiponectin, which constitutes a novel therapeutic strategy for obese children with CPP. Adipokines function as signaling molecules for pathways related to metabolism and immunity (87). Adiponectin, a multifunctional adipokine with anti-inflammatory activity, improves insulin sensitivity and helps maintain vascular endothelial function, but its level is often decreased in obesity (88). In contrast, leptin and resistin are generally regarded as pro-inflammatory mediators and are closely related to insulin resistance and chronic inflammation (88). The dual regulation therapy of the kidney and spleen may therefore restore the balance among adipokines. The adiponectin/leptin (LEP/ADPN) ratio is a critical indicator of metabolic status (88), and TCM treatment that increases adiponectin while decreasing leptin and resistin levels may improve insulin signaling, reduce oxidative stress, and regulate immune cell activity, thereby relieving metabolic inflammation. In obesity-related CPP, inflammatory signals arising from adipose tissue dysfunction can directly or indirectly disturb the HPG axis, thereby promoting early pubertal activation. Given that the NEI network continuously coordinates energy balance, immune responses, and neuroendocrine function, normalizing the LEP/ADPN ratio may offer therapeutic benefits.

From a biomarker perspective, leptin, adiponectin, and the LEP/ADPN ratio can identify the metabolic subtype of CPP and help monitor metabolic improvement during treatment, particularly in children who are overweight or obese. A recent systematic review and meta-analysis reported that girls with CPP generally showed higher circulating leptin levels and lower adiponectin levels compared to normal controls, whereas irisin levels did not differ significantly (28). This pattern is consistent with the biological characteristics of obesity-related CPP, where hyperleptinemia reflects increased adiposity and possible leptin resistance, while reduced adiponectin indicates weakened anti-inflammatory and insulin-sensitizing capacity. Therefore, these indicators may help distinguish children with CPP accompanied by metabolic inflammation from healthy children or from CPP patients without obvious metabolic disturbance, although they should be used as complementary biomarkers rather than independent diagnostic criteria. In terms of monitoring therapeutic efficacy, adipokines may be more useful for evaluating metabolic and inflammatory responses as opposed to HPG axis suppression. Interestingly, GnRH agonist therapy has no significant impact on the serum leptin and adiponectin levels in girls with CPP, suggesting that these markers are not equivalent to conventional endocrine outcomes such as LH, FSH, estradiol, or bone-age progression (29). However, another clinical study found that higher baseline leptin levels and a higher LEP/ADPN ratio were associated with greater weight gain during GnRH agonist treatment, indicating that adipokine imbalance may predict treatment-associated metabolic risk (30). Furthermore, the LEP/ADPN ratio has been shown to outperform leptin or adiponectin alone in identifying metabolic risk in pediatric subjects; metabolic abnormalities correspond to increased leptin levels and LEP/ADPN ratio, and decreased adiponectin levels (31). Therefore, future CPP studies should consider dynamic assessment of leptin, adiponectin, and the LEP/ADPN ratio along with BMI standard deviation score, waist circumference, insulin resistance indices, lipid profile, GnRH stimulation results, sex hormone levels, and bone-age advancement. This combined evaluation may help determine whether kidney-spleen regulating therapy can improve both pubertal progression and the metabolic-inflammatory background that contributes to obesity-related CPP.

In summary, dual regulation of the kidney and spleen may improve glucose and lipid metabolism, reduce insulin resistance, and regulate beneficial adipokines such as adiponectin. At the same time, it may create a more stable internal environment for the HPG axis by lowering systemic chronic low-grade inflammation and blocking the inflammatory pathway involved in obesity-induced precocious puberty. This reflects the holistic concept of TCM and the preventive treatment principle in managing complex metabolic reproductive comorbidities.

## 6 Challenges, prospects and future research directions

### 6.1 Current challenges

The objectification and standardization of TCM syndrome diagnosis remain major challenges in current research. These difficulties directly affect the consistency of clinical studies and the accuracy of therapeutic evaluation. At present, TCM syndrome identification mainly depends on the clinical experience of physicians, and diagnosis is based on inspection, auscultation and olfaction, inquiry, and palpation. Although this approach reflects the holistic thinking and individualized treatment principles of TCM, it also has limitations given the subjective nature of diagnostic judgments. As a result, different physicians may classify the same patient into different syndrome types, which reduces the homogeneity of study populations and weakens the objectivity of efficacy assessment. Likewise, the diagnosis of diabetes and prediabetes based on pulse and tongue characteristics often depends on the physician’s skill, training, and clinical experience, which can result in inconsistent syndrome classification across different hospitals, researchers, or clinical centers. These variations not only confound the comparison of findings between studies and lowers reproducibility but also create challenges for randomized controlled trials (RCTs) based on TCM syndromes. RCTs require clear diagnostic criteria, standardized interventions, and uniform outcome evaluation, while TCM emphasizes syndrome differentiation and individualized treatment. If enrolled patients have different baseline syndrome characteristics because of inconsistent diagnosis, the actual therapeutic effect may be hidden, exaggerated, or wrongly interpreted. Therefore, unclear syndrome classification may reduce the reliability and generalizability of clinical findings.

To overcome these problems, researchers are increasingly focusing on the development of clinical practice guidelines and expert consensus using evidence-based medicine. For several diseases, including recurrent spontaneous abortion, cervical spondylosis, and fibromyalgia syndrome, professional committees have developed diagnosis and treatment guidelines for TCM or integrated traditional Chinese and Western medicine. These guidelines usually include common syndrome classification standards and aim to unify clinical diagnosis and treatment. The development of these guidelines involves literature reviews, expert consultation, and Delphi consensus methods, which can improve the stability, reproducibility, and clinical usefulness of syndrome differentiation. However, the Delphi method for TCM syndrome diagnosis also has limitations, such as inconsistent expert agreement and insufficient transparency in reporting. This underscores the need for a more unified and clearly reported methodology. In addition, researchers have attempted to establish quantitative diagnostic standards for syndrome elements in some diseases. For example, quantitative criteria for water-dampness syndrome and damp turbidity syndrome have been developed through literature analysis and multiple rounds of expert consultation to diagnose idiopathic membranous nephropathy. These criteria were further evaluated through clinical diagnostic tests to assess their sensitivity and specificity. These studies provide a useful model for enhancing the objectivity of TCM syndrome diagnosis. Future research on CPP should focus on establishing clearer and more measurable standards for kidney-spleen syndrome differentiation while preserving the core principles of TCM.

### 6.2 Future research directions and integration strategies

#### 6.2.1 Prospective development and validation of syndrome-specific biomarkers

Actionable biomarker development should begin with multi-center prospective recruitment of treatment-naive children with biochemically confirmed CPP. The three proposed patterns – kidney yin deficiency with hyperactive ministerial fire, spleen deficiency with phlegm-dampness, and a mixed kidney-spleen pattern – should be assigned independently by two TCM clinicians in a blinded manner using prespecified criteria, with adjudication of discordant cases. Sampling should be stratified by sex, age, adiposity, pubertal tempo, bone-age advancement, etiology, and subsequent GnRH agonist use. Candidate panels should integrate basal and stimulated LH, the LH/FSH ratio, estradiol or testosterone, IGF-1, bone-age and pelvic indices or testicular volume with leptin, adiponectin, LEP/ADPN ratio, fasting insulin or the homeostasis model assessment of insulin resistance, inflammatory cytokines, microbial functions, SCFAs, and bile-acid species. Proteomic-metabolomic and microbiome-metabolome analyses of biological samples from children with CPP have identified lipid, immune, and microbial-metabolic candidates (89, 90), but have not established TCM syndrome specificity. Discovery signatures should therefore be validated externally, adjusted for diet, obesity, recent antibiotics or probiotics, and analytical batch effects, and evaluated for calibration, discrimination, clinical utility, and longitudinal prediction of response to standardized kidney-spleen therapy.

Future studies should combine modern biological techniques with TCM diagnostic reasoning and define genetic and epigenetic indices. Causal DNA variants, parent-of-origin imprinting, tissue-specific gene expression, DNA methylation, and RNA m6A modification are not interchangeable biomarkers. MKRN3 sequencing should be accompanied by parental-origin analysis because a pathogenic variant on the expressed paternal allele has different functional implications from the same variant on the silenced maternal allele (5). The relevant experimental readouts for FTO are tissue-specific expression, and m6A modification of candidate transcripts such as Bdnf; the available preclinical evidence does not establish a causal FTO variant in human CPP (10). Multi-omics analyses can also be used to identify the molecular signatures associated with kidney- or spleen-related syndrome patterns, although the association should not be extrapolated to actionable therapeutic targets. Furthermore, studies conducted on TCM prescriptions with kidney and spleen-regulating activity should clarify the experimental readouts – downstream endocrine-metabolic improvement or direct regulation of a candidate mechanism. Claims of direct action on MKRN3 should be supported by assessment of allele-specific expression or the MKRN3-MBD3/TET2 axis, whereas FTO-related conclusions should be based on FTO abundance, Bdnf m6A status, and perturbation or rescue experiments. Until such evidence is available, changes in KISS1/GnRH, gonadotropins, inflammatory markers, or metabolic phenotypes should be described as downstream effects, not correction of genetic or epigenetic lesions.

#### 6.2.2 Targeted intervention studies of the gut microbiota-bile acid axis

Human studies have identified distinct fecal microbial and metabolite profiles in girls with CPP (55, 90). Furthermore, glycodeoxycholic acid delayed puberty in a high-fat diet-induced CPP rat model and altered microbiota-metabolite and hypothalamic sirtuin 1 (SIRT1)/Kiss1 expression (65). On the other hand, elevated muricholic acids in female CPP subjects and modelled mice implicated Takeda G protein-coupled receptor 5 (TGR5) and downstream phosphoinositide 3-kinase (PI3K)/protein kinase B (Akt)/mechanistic target of rapamycin (mTOR) signaling in GnRH neurons (91). Furthermore, a study combining observational pediatric analysis, danazol-treated rat model, and fecal microbiota transfer experiments suggested microbiome-mediated modulation of pubertal timing by glycyrrhizin (92). These findings identify testable targets, but do not establish the pediatric efficacy or long-term safety of bile acids, probiotics, or herbal constituents, thus warranting a stepwise translational pathway. Longitudinal cohorts should pair shotgun metagenomics with targeted serum and fecal bile acid and SCFA profiling at baseline and 3, 6, and 12 months, while recording diet, adiposity, antibiotics, probiotics, and GnRHa exposure. Standardized kidney-spleen prescriptions or defined constituents should then be tested in CPP models using antibiotic depletion or fecal microbiota transfer, along with perturbation of SIRT1/Kiss1 or TGR5 signaling to demonstrate microbiota dependence and target engagement. Small adjunctive pediatric trials should be conducted after the requirements for reproducibility, dose-response, pharmacokinetics, and developmental toxicology are satisfied to evaluate a standardized formula, microbiota-directed nutrition, or a defined synbiotic. Prespecified endpoints should include stimulated LH, sex steroids, bone-age progression, microbial function, bile-acid target engagement, and adverse events; direct administration of experimental bile acids should not proceed to pediatric testing without dedicated safety data.

### 6.3 Long-term safety and clinical risk management in children with CPP

Since children with CPP are in a critical period of growth and endocrine maturation, the long-term safety of kidney-spleen regulating prescriptions should be considered with the same importance as endocrine efficacy. Existing systematic reviews of herbal medicine for idiopathic CPP and female precocious puberty suggest that Chinese herbal medicine may improve sexual characteristics, hormone levels, or bone-age-related outcomes, although the evidence of safety remains limited by small sample sizes, short observation periods, incomplete adverse-event reporting, and insufficient follow-up to final height or late puberty (32, 33). Therefore, the relatively acceptable safety profile reported in current clinical studies should be interpreted as preliminary rather than definitive evidence of long-term safety.

The formulas discussed in this review, including modified Zhibai Dihuang pills combined with Liujunzi decoction and modified Danzhi Xiaoyao powder combined with Erchen decoction, are mainly administered orally and often need repeated or prolonged administration according to syndrome evolution. In general clinical practice, the most common adverse reactions associated with oral TCM formulations are gastrointestinal symptoms, such as abdominal discomfort, nausea, vomiting, loose stool, diarrhea, poor appetite, and taste intolerance. A systematic review of adverse events in randomized trials of Chinese herbal medicine reported an overall adverse-event incidence of 10.8%, with gastrointestinal disorders being the most common category, followed by liver and biliary disorders. In addition, the incidence of adverse events was higher in child participants than in adults (93). This finding is particularly relevant to CPP because pediatric patients have immature drug metabolism, changing body weight, and higher sensitivity to dosage errors or prolonged exposure.

Although the specific kidney-spleen regulating formulas discussed in this article have not been consistently associated with severe liver or kidney injury in CPP studies, potential hepatorenal toxicity should be carefully considered. Yin-nourishing and fire-purging prescriptions may contain Zhimu (*Anemarrhena asphodeloides* Bunge) and Huangbai (*Phellodendron chinense* Schneid). Experimental and pharmacological reviews indicate that active constituents of *Anemarrhena*, especially timosaponin AIII, require further toxicological evaluation as hepatotoxicity has been observed in experimental settings and may involve oxidative stress and bile-acid transporter disturbance (94). Phellodendri Cortex contains protoberberine alkaloids such as berberine; pharmacokinetic studies suggest that related compounds may localize to the liver and kidney, which warrants caution in susceptible populations (95). These data do not suggest a lack of safety but emphasize the need for rational dosing, standardized processing, and laboratory monitoring during long-term pediatric treatment.

Notably, Chinese herbal medicine should not be regarded as safe for the liver simply because it is derived from natural products. LiverTox notes that only a limited number of Chinese and Asian herbal products have been clearly linked to clinically apparent liver injury; nevertheless, variability in formula composition, mislabeling, contamination, and concomitant drug use can complicate causality assessment (96). In a Chinese study on pediatric drug-induced liver injury, TCM formulations or decoctions accounted for a significant proportion of suspected cases, and children with TCM-related liver injury showed higher bilirubin levels and longer prothrombin time compared to those with Western medicine-related liver injury (97). Although this evidence does not specifically implicate the formulas used for CPP, it indicates that liver function should be actively monitored when herbal prescriptions are used over several months in children.

For clinical application, several safety principles should be emphasized. First, prescriptions should be individualized according to syndrome differentiation, age, body weight, body surface area, digestive tolerance, and baseline liver and kidney function. Second, raw or improperly processed toxic herbs should be avoided, and all components should comply with pharmacopeial processing and quality-control standards. Third, concurrent use of multiple patent medicines, health supplements, or unsupervised herbal products should be discouraged to reduce the risk of duplicated ingredients and herb-drug interactions, particularly when GnRH analogues or other endocrine therapies are used. Fourth, baseline and periodic monitoring should include alanine aminotransferase, aspartate aminotransferase, alkaline phosphatase, gamma-glutamyl transferase, total bilirubin, albumin, serum creatinine, blood urea nitrogen, estimated glomerular filtration rate, and urinalysis for prolonged treatment. Finally, treatment should be suspended and reassessed in case of persistent gastrointestinal intolerance, rash, jaundice, dark urine, unexplained fatigue, abnormal bleeding tendency, elevated transaminases, bilirubin increase, or deterioration of renal function.

Long-term safety should be investigated through a prospective registry embedded in multicenter trials. Assessments should occur before treatment, at 4–8 weeks during treatment, every 3 months during therapy, at discontinuation, and at 6, 12, and 24 months thereafter, with follow-up extended to near-final or final height and post-treatment pubertal recovery. Core outcomes should include adverse events, adherence, height velocity, BMI and body composition, Tanner stage, bone age, basal or stimulated gonadotropins, sex steroids, glucose and lipid metabolism, liver and kidney tests, menstrual recovery or testicular development, and age-appropriate quality-of-life and psychological measures. Long-term GnRHa cohorts demonstrate the feasibility of tracking adult height, menarche, metabolic status, and reproductive outcomes and provide relevant comparator endpoints (98, 99). Exposure data should be product-specific: botanical identity, processing method, batch fingerprint, contaminants, dose per kilogram, cumulative exposure, adherence, and concomitant medicines or supplements should be recorded. Analyses should compare standardized kidney-spleen therapy plus GnRHa with GnRHa alone, stratify results by sex, obesity, and syndrome pattern, and incorporate independent causality assessment and data-safety monitoring. This framework would distinguish formula-related toxicity from background symptoms and provide the longitudinal evidence required before prolonged pediatric use can be recommended.

## 7 Conclusion

Pediatric CPP is closely associated with kidney and spleen dysfunction in TCM. Dual kidney-spleen regulation therefore represents an important therapeutic principle that is also related to the NEI described in modern medicine. Based on the evidence discussed in this review, dual regulation of the kidney and spleen should not be considered a single-target treatment. Instead, it may work as a multi-level and holistic regulatory approach that simultaneously acts on several biological pathways. This therapeutic strategy may help suppress premature activation of the HPG axis, regulate immune-inflammatory responses, reduce neural stress, and improve the overall imbalance of the NEI network. Immune regulation appears to be one of the key mechanisms involved in this process. By restoring immune cell balance, reducing chronic inflammatory activity, and improving immune-neuroendocrine communication, dual kidney-spleen regulation may help limit immune-mediated pathological changes associated with CPP. Therefore, the management of CPP should focus not only on controlling the HPG axis, but also the wider interactions among the endocrine, immune, nervous, inflammatory, and metabolic systems.

Clinical reports suggest that syndrome differentiation and treatment based on dual kidney-spleen regulation may have useful effects and acceptable short-term safety in CPP management. However, these findings should be interpreted carefully and objectively. While the holistic and individualized nature of TCM has clear value, modern medicine also plays an important role in accurate diagnosis, hormone evaluation, and targeted clinical intervention. Therefore, the future direction should be integration rather than replacement. Systems biology, omics technology, cytokine network analysis, and gut microbiota research should be used to further explain how kidney-spleen regulation affects the NEI network and how traditional syndrome differentiation can be connected with modern biological mechanisms.

The present review proposes that the hypothalamic kisspeptin/GPR54/GnRH signaling cascade should be prioritized as the most promising mechanistic target for in-depth validation. Compared with broader descriptions of neuro-endocrine-immune regulation, this pathway is directly positioned at the central switch of pubertal onset and is supported by available experimental evidence showing that a TCM formula can downregulate kisspeptin/GPR54-related signaling and reduce GnRH secretion in hypothalamic GnRH neuronal cells (46). Therefore, future *in vitro* and *in vivo* studies should use hypothalamic neuronal models, CPP animal models induced by high-fat diet or endocrine disruptors, and genetic or pharmacological manipulation of GPR54/GnRH signaling to determine whether kidney-spleen regulating prescriptions exert reproducible inhibitory effects on premature HPG axis activation. Among possible component-level candidates, timosaponin-related constituents from *A. asphodeloides* and berberine-type alkaloids from *P. chinense* may be considered exploratory compounds as they represent key constituents of yin-nourishing and fire-purging herbs; however, their direct anti-CPP activity remains to be verified, and should be assessed along with endocrine efficacy, hypothalamic pathway readouts, pharmacokinetics, and pediatric safety parameters (94, 95). To summarize, clinical translation should start with external validation of syndrome-specific biomarker panels, followed by causal and target-engagement studies of microbiota-bile acid pathways before pediatric intervention, and further progress to registry-based surveillance through final height and post-treatment pubertal recovery. Multicenter trials can then determine the CPP phenotypes and developmental stages that benefit from adjunctive kidney-spleen therapy, along with the long-term risk-benefit profile.
